# The Association of SARIFA with Poor Prognosis and Aggressive Tumor Phenotypes in Cancers: A Meta-Analysis

**DOI:** 10.3390/jcm15176830

**Published:** 2026-09-03

**Authors:** Jiaqi Lao, Guangwei Tian, Lin Guan

**Affiliations:** 1 Department of Gastroenterology, The First Hospital of China Medical University, No. 155 North Nanjing Street, Heping District, Shenyang 110001, China; laojq1121@163.com; 2 The First Clinical College, China Medical University, No. 77 Puhe Street, Shenbei New District, Shenyang 110122, China; 3 Department of Radiation Oncology, The First Hospital of China Medical University, No. 155 North Nanjing Street, Heping District, Shenyang 110001, China; tgwcmu8901@126.com

**Keywords:** meta-analysis, prognosis, overall survival, tumor staging

## Abstract

**Background**: SARIFA (Stroma Areactive Invasion Front Areas) is an emerging histopathological biomarker defined by direct tumor–adipocyte contact at the invasive front. Preliminary studies suggest its association with poor prognosis, but evidence remains fragmented across cancer types. This meta-analysis aims to systematically evaluate the prognostic value of SARIFA for overall survival (OS) and its correlation with aggressive tumor phenotypes. **Methods**: Following PRISMA guidelines, we searched PubMed, Embase, Web of Science, and Cochrane up to December 2025. Studies reporting hazard ratios (HRs) for OS or odds ratios (ORs) for high-grade tumors related to SARIFA status were included. Data were pooled using Stata 12.0, and heterogeneity was assessed using I^2^ statistics. Subgroup, sensitivity, and publication bias analyses were performed. **Results**: Ten studies (6881 patients) showed that SARIFA positivity was significantly associated with worse OS (HR = 1.52, 95% CI: 1.42–1.63, I^2^ = 0.0%). Eleven studies (6847 patients) revealed that SARIFA positivity correlated with advanced tumor stage: pT (OR = 5.49), pN (OR = 2.80), pM (OR = 2.03), AJCC stage (OR = 4.04), and higher pathological grade (OR = 2.36). Heterogeneity was low for OS but substantial for pT (I^2^ = 87.4%), pM (I^2^ = 70.5%), and AJCC (I^2^ = 72.0%). Publication bias was absent for OS but present in pT- and AJCC-staging analyses. Trim-and-fill correction for pT indicated that statistical significance disappeared after imputing two missing studies (*p* changed from <0.001 to 0.450), suggesting that this association is not robust. Evidence for pancreatic and prostate cancers remains limited, each represented by only one study. **Conclusions**: SARIFA positivity is a strong predictor of poor overall survival and is significantly associated with aggressive tumor phenotypes, including advanced pT, pN, pM, AJCC stage, and higher pathological grade, supporting its potential as a practical prognostic biomarker in solid tumors.

## 1. Introduction

Cancer is a major public health issue worldwide. It is estimated that in 2022, colorectal cancer, prostate cancer, and gastric cancer became the three most common cancers after lung cancer and breast cancer, accounting for 9.6%, 7.3%, and 4.9% of all cancer cases globally, respectively. Meanwhile, colorectal cancer is the second leading cause of cancer death after lung cancer [1]. Currently, prognosis assessment mainly relies on the TNM staging system. However, significant differences in clinical outcomes have been found among colorectal cancer patients even at the same stage [2]. Therefore, there is an urgent need for reliable and efficient prognostic biomarkers to stratify risk, guide treatment decisions, and identify high-risk patients.

The invasive front represents a biologically active interface where tumor cells interact with surrounding stromal, immune, and adipose compartments. Morphological characteristics at this interface may therefore capture aspects of tumor aggressiveness that are not fully represented by conventional anatomical staging. Against this backdrop, studies have proposed a novel prognostic marker, Stroma Areactive Invasion Front Areas (SARIFA), a morphological marker evaluated on conventional hematoxylin–eosin (H&E)-stained sections. SARIFA is defined as the direct contact area between tumor glands/tumor cell clusters (at least five cells) and the inconspicuous surrounding adipose tissue in the invasive front [3]. Studies have shown that adipocytes and lipids can create a microenvironment conducive to tumor growth [4,5].

Preliminary research indicates that SARIFA is closely associated with poor prognosis in cancer patients. For instance, studies have shown that in gastric cancer, SARIFA positivity is an independent negative predictor of overall survival [6]. Similarly, in colorectal cancer, SARIFA has been confirmed to be significantly associated with more advanced TNM staging, lymphovascular invasion, and shorter survival [7,8]. Recent research evidence further links the positive status of SARIFA to aggressive biological behavior in pancreatic cancer and prostate cancer [9,10]. Beyond the tumor types mentioned above, recent research on SARIFA has been extended to cutaneous melanoma. Melanoma-adapted SARIFA was found to correlate with lymphovascular invasion and microsatellitosis [11]. The assessment of SARIFA does not rely on complex molecular testing but can be performed using routine histological sections, which endows it with great potential for clinical translation.

Recent mechanistic studies have substantially advanced our understanding of SARIFA biology. Evidence from two large cohort studies demonstrated that SARIFA positivity in colorectal cancer is associated with reduced densities of antitumor immune cells including CD3^+^ T cells, CD66b^+^ granulocytes, and M1-like macrophages, alongside polarization of macrophages toward a protumorigenic M2-like phenotype [12]. Additional findings have indicated that SARIFA positivity provides additive prognostic value across molecular subgroups of colorectal cancer. SARIFA-positive tumors were more frequently observed in BRAF-mutant and BRAF-mutant/mismatch repair-proficient cases. Notably, the survival benefit from adjuvant therapy was confined to SARIFA-positive patients, suggesting that SARIFA status may hold predictive value for treatment response [13]. At the molecular level, accumulating evidence has linked SARIFA positivity to elevated expression of the plasmin/plasminogen activator system (uPA/PAI-1) and its downstream matrix metalloproteinases. Digital spatial profiling further revealed upregulation of PLAU in tumor cells and coordinated upregulation of PLAUR in adjacent stromal cells within SARIFA regions, suggesting a potential ligand–receptor interaction between the two compartments. Collectively, these observations not only deepen our understanding of the biological underpinnings of SARIFA but also implicate the uPA/PAI-1 axis as a candidate therapeutic vulnerability [14].

However, current evidence on the prognostic value of SARIFA remains fragmented across cancer types, and the stromal composition, lipid metabolism, immune microenvironment, and invasion patterns vary substantially among different malignancies. Moreover, the sample sizes and conclusions of individual studies are heterogeneous. Whether SARIFA can serve as a universal and robust prognostic indicator across multiple cancer types has not yet been systematically and quantitatively established. In addition, the association between SARIFA status and tumor pathological features, such as tumor grade, has not been comprehensively summarized. Therefore, rather than assuming a pan-cancer universal effect, this meta-analysis was designed to evaluate the overall association between SARIFA and survival, while critically examining heterogeneity and the robustness of the evidence across cancer types (colorectal, gastric, pancreatic, and prostate) and pathological endpoints. We also analyzed the association between SARIFA and advanced clinicopathological characteristics, including pT, pN, pM, AJCC stage, and pathological grade, to provide a comprehensive evidence base for future research and clinical translation.

## 2. Materials and Methods

This study strictly adhered to the internationally recognized PRISMA guidelines [15] (Supplementary Table S1). The research plan was registered on the PROSPERO platform (registration number: CRD420251272196). The review was conducted in accordance with the registered protocol. No amendments were made to the protocol after registration. The scientific objective of this study is to systematically evaluate the prognostic value of the positive state of Stroma Areactive Invasion Front Areas (SARIFA) in the tumor microenvironment on the overall survival (OS) of patients with various solid tumors through quantitative synthesis methods, and simultaneously analyze the potential association between the positive SARIFA and advanced tumor pathological features, thereby providing high-level evidence-based medical evidence for cross-cancer biological marker research.

### 2.1. Search Strategy

To comprehensively identify all relevant studies, we developed a systematic and repeatable search strategy. We conducted a systematic search in several databases: PubMed, Embase, Web of Science, and Cochrane. The search time range was from the inception date of each database to December 2025. We used both subject terms and free words to construct the search terms, ensuring high recall and precision. The search strategy was determined through multiple pre-searches and optimizations. To ensure transparency and reproducibility, the detailed search strategies for all four databases (PubMed, Embase, Web of Science, and the Cochrane Library) are available in Supplementary Table S2. All retrieved literature citations were imported into Zotero (Version 10.0), a literature management software, and duplicate records were automatically removed.

### 2.2. Inclusion and Exclusion Criteria

The inclusion criteria were as follows: (a) the subjects of the studies included patients with any cancer; (b) the SARIFA status of the patients was evaluated pathologically (positive, negative, or unassessable); (c) the studies reported various survival information related to the SARIFA status, such as the hazard ratio (HR) and its 95% confidence interval of indicators like overall survival (OS) or disease-specific survival (DSS), or provided sufficient information to calculate this data (such as the Kaplan–Meier survival curve); (d) the association between SARIFA status and high-grade tumors was reported, and the odds ratio (OR) and its 95% confidence interval, or derived contingency table data, were provided.

### 2.3. Identification and Eligibility Assessment

**Figure 1:**
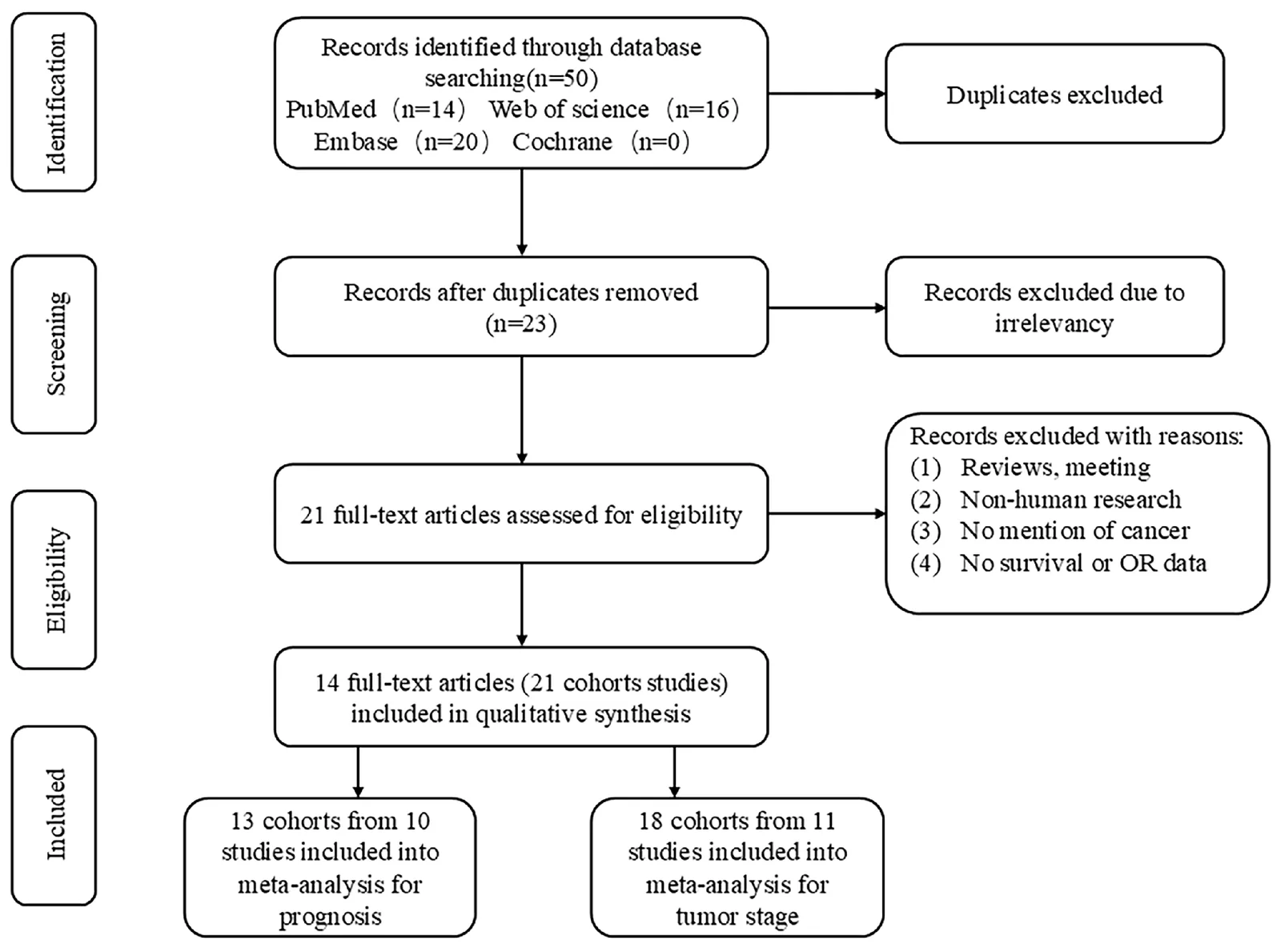
Flow diagram of the study selection procedure.

Two reviewers independently screened the titles and abstracts of all records identified through the electronic searches. Full-text articles were subsequently retrieved and independently assessed for eligibility according to the predefined inclusion and exclusion criteria. Any disagreements between the two reviewers were resolved through discussion, and consensus was reached when necessary. If consensus could not be achieved, a third reviewer was consulted to make the final decision. The study selection process is summarized in the PRISMA flow diagram (Figure 1).

The exclusion criteria were as follows: (a) the research types were case reports, reviews, commentaries, conference summaries, meta-analyses, and basic scientific research (such as cell or animal experiments); (b) the research was repetitive; (c) data were missing or incorrect; (d) only SARIFA was studied, without including survival information, odds ratios, or other data.

### 2.4. Data Extraction and Quality Evaluation

Data were independently extracted by two reviewers using a predesigned and standardized data extraction form. The following information was collected: first author, year of publication, country, study design, data source, cancer type, sample size, number of SARIFA-positive and SARIFA-negative patients, patient characteristics, clinicopathological characteristics, definition of SARIFA positivity, survival outcomes, effect estimates, and variables adjusted for in multivariable analyses. When discrepancies occurred during data extraction, these were resolved through discussion and consensus. When relevant data were unclear or unavailable, the corresponding authors were contacted whenever possible. If the required information could not be obtained, the study was included only in analyses for which sufficient data were available. No assumptions were made to replace unavailable outcome data.

The two researchers used the Newcastle–Ottawa scale to evaluate the methodological quality of the included cohort studies. This scale consists of 8 items, covering three dimensions: “Selection of study subjects” (4 items), “Comparability between groups” (1 item), and “Outcome assessment” (3 items). The maximum total score is 9 stars [16] (Table 1).

### 2.5. Eligibility for Each Synthesis

Studies were included in each quantitative synthesis only when they reported sufficient data for the corresponding outcome. Studies reporting HRs and 95% CIs for OS were included in the survival meta-analysis. Studies reporting contingency data or effect estimates for the association between SARIFA status and clinicopathological characteristics were included in the corresponding analyses of pT, pN, pM, AJCC stage, and pathological grade. Because not all studies reported all outcomes, the number of studies included varied across different syntheses.

### 2.6. Statistical Analysis

This study included a meta-analysis using STATA 12.0 software (STATA, College Station, TX, USA). The primary outcome was overall survival (OS), defined as the time from diagnosis or treatment to death from any cause, according to the definitions used in the original studies, and the risk ratio and its 95% confidence interval of the impact of SARIFA positivity on OS were evaluated by combining various effect sizes. Secondary outcomes included the associations between SARIFA status and clinicopathological characteristics, including pathological tumor stage (pT), pathological nodal stage (pN), pathological metastatic stage (pM), AJCC stage, and pathological grade. For each study, we collected the frequencies of SARIFA-positive and SARIFA-negative cases stratified by high- and low-grade tumor groups, and computed ORs to evaluate whether SARIFA positivity was associated with a higher likelihood of advanced clinicopathological features. Heterogeneity in the study was evaluated using the I^2^ statistic [21]. I^2^ ≤ 25% was considered low heterogeneity, 25% < I^2^ ≤ 50% was moderate heterogeneity, and I^2^ > 50% was high heterogeneity. Based on the heterogeneity results, the effect model was selected. When *p* < 0.10 or I^2^ > 50%, significant heterogeneity was indicated, and a random-effect model was used; otherwise, a fixed-effect model was used. To explore the source of heterogeneity, subgroup analyses were conducted by cancer type, region, sample size, data type, data source (single-center/multi-center), and SARIFA definition. Begg’s test and Egger’s test were used to evaluate publication bias.

### 2.7. Certainty of Evidence

The certainty of evidence for each outcome was assessed using the Grading of Recommendations Assessment, Development and Evaluation (GRADE) approach. The certainty of evidence was evaluated according to the risk of bias, inconsistency, indirectness, imprecision, and publication bias. The certainty of evidence was categorized as high, moderate, low, or very low.

### 2.8. Generative AI Usage

During manuscript preparation, the DeepSeek large-language model was used for language translation and English linguistic polishing. No GenAI tool was involved in study design, literature screening, data extraction, statistical analysis, or interpretation of results. All AI-assisted outputs were fully reviewed, revised, and edited manually by the authors, who take full responsibility for the entire content of this manuscript.

## 3. Results

### 3.1. Research Characteristics

The search results included 50 studies across four databases: PubMed (*n* = 14), Web of Science (*n* = 16), Embase (*n* = 20), and Cochrane (*n* = 0). After removing the duplicate literature and screening based on titles and abstracts, 14 studies were retained for meta-analysis, involving 8438 patients across 21 cohorts. Figure 1 shows the flowchart of the study screening process.

The studies were conducted in five regions: Finland (*n* = 1), Germany (*n* = 9), the Netherlands (*n* = 2), Hungary (*n* = 1), and the UK (*n* = 1). The tumors involved included colorectal cancer (*n* = 7) [3,7,8,12,13,17,18], gastric cancer/esophageal gastric cancer (*n* = 5) [6,19,20,22,23], pancreatic ductal adenocarcinoma (*n* = 1) [9], and prostate cancer (*n* = 1) [10]. Table 1 presents the characteristics of the included studies (Table 1).

**Figure 2:**
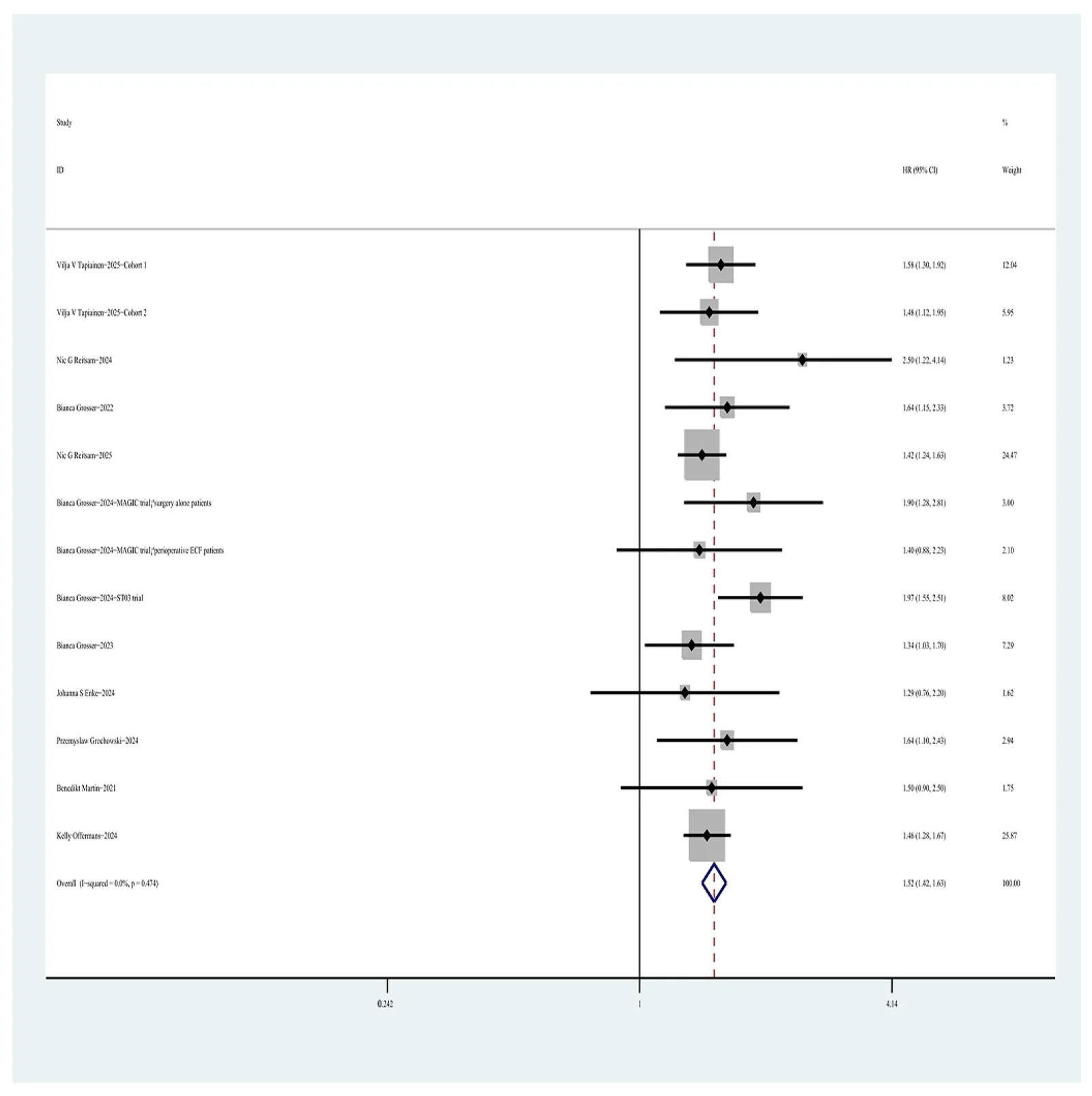
Forest plot of the association between SARIFA positivity and overall survival (OS) [3,6,9,10,12,13,17,18,19,20].

There were 10 studies on the relationship between SARIFA positivity and overall survival (OS), involving 13 cohorts of 6881 patients. The meta-analysis results showed that HR = 1.52 (95% CI: 1.42–1.63, *p* = 0.474, I^2^ = 0.0%), indicating that SARIFA positivity was a strong indicator of poor prognosis for patients, associated with a significant increase in mortality risk. Moreover, heterogeneity among these 13 cohorts was not significant, and the studies were highly homogeneous. Three of the cohorts had confidence intervals including 1, suggesting that the prognostic effect of SARIFA did not reach statistical significance. This might have been due to small sample size or changes in treatment methods affecting the impact of SARIFA (Figure 2).

### 3.2. Subgroup Analysis

**Figure 3:**
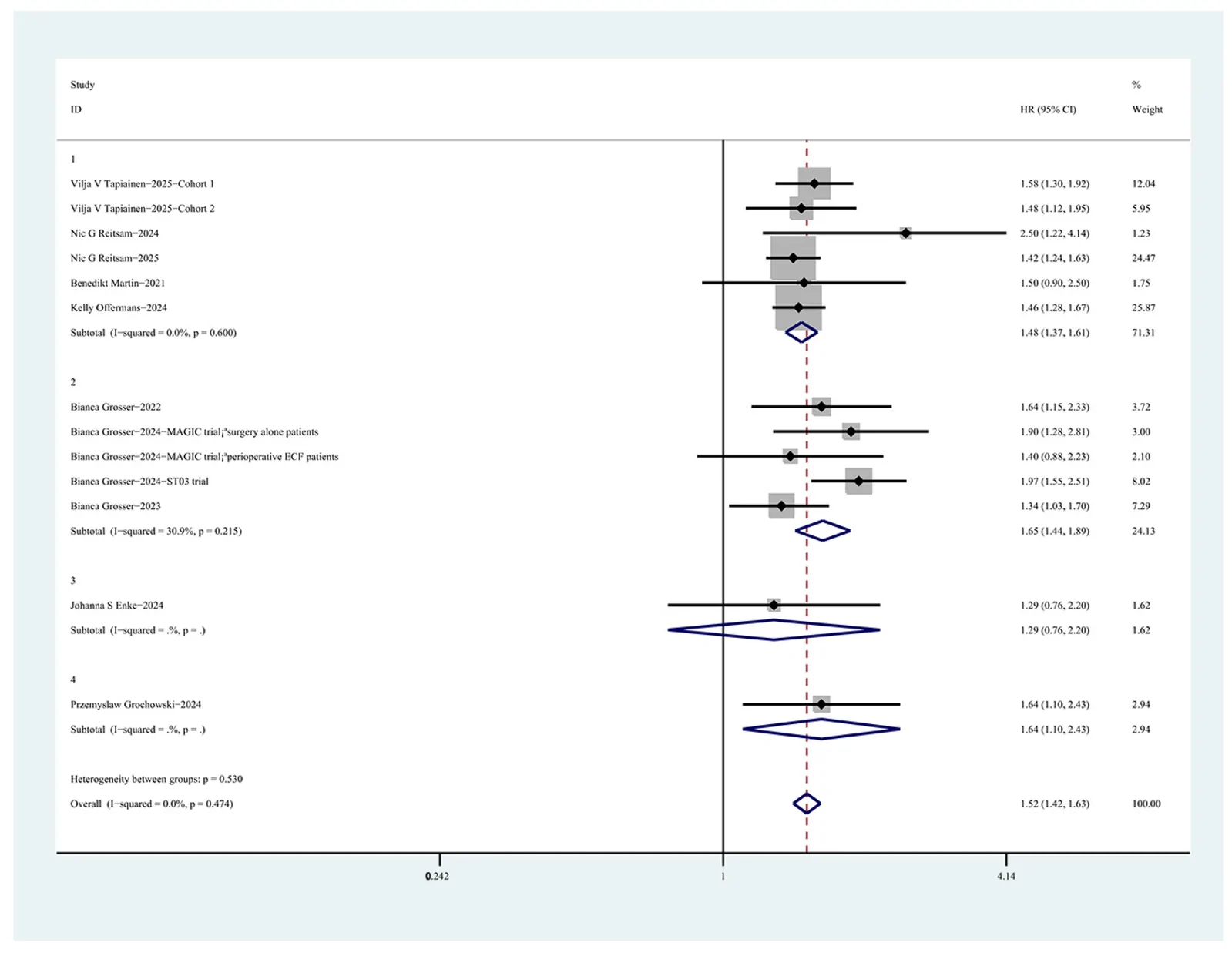
Subgroup analysis results. Subgroup analysis of OS by cancer type. HRs and 95% CIs are presented for colorectal, gastric/oesophagogastric, pancreatic, and prostate cancers. Heterogeneity within subgroups is indicated by I^2^ [3,6,9,10,12,13,17,18,19,20].

**Figure 4:**
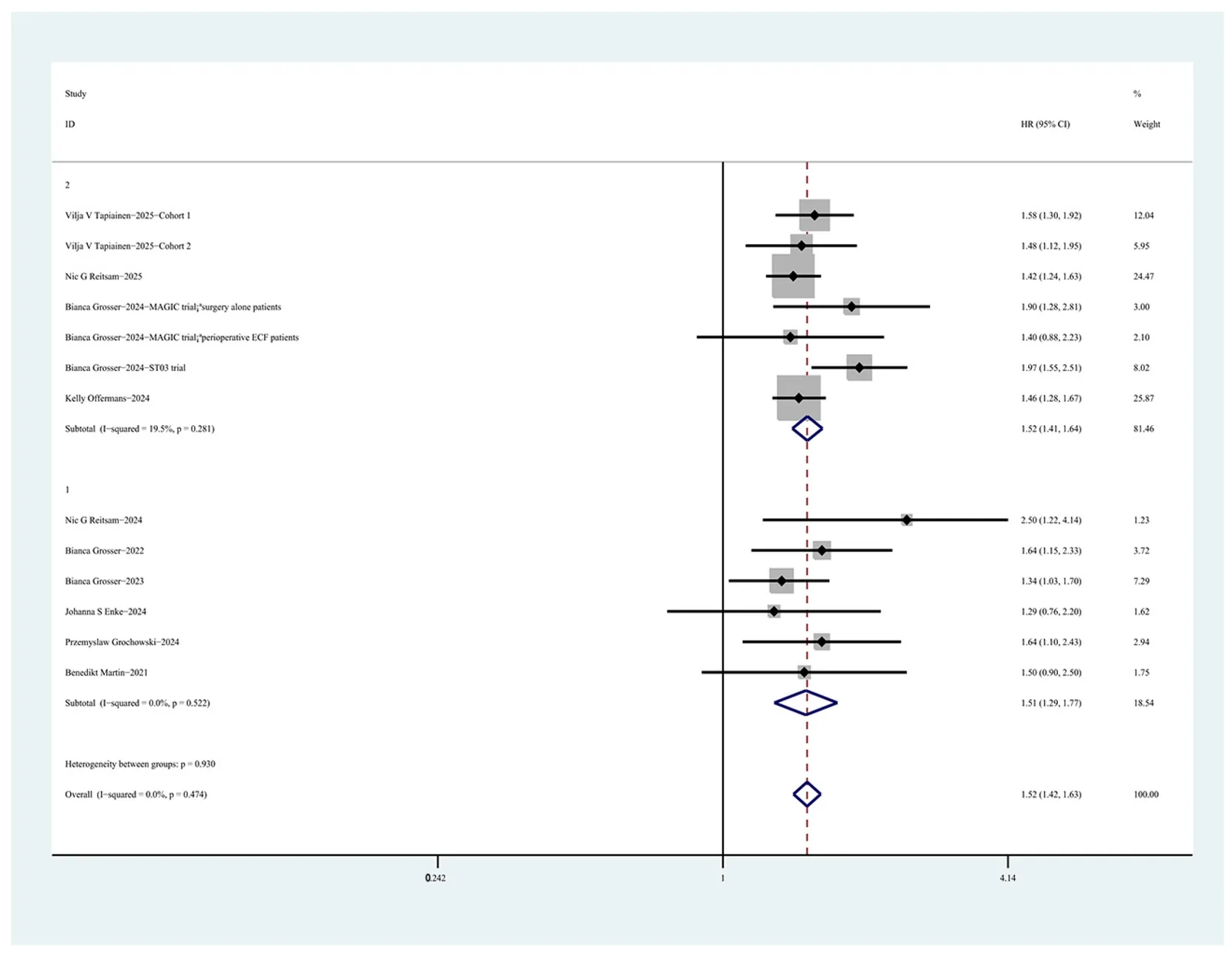
Subgroup analysis results. Subgroup analysis of OS by geographic region (Germany vs. other European countries). The pooled HRs are comparable, suggesting that geographic factors did not substantially affect the OS association [3,6,9,10,12,13,17,18,19,20].

In the subgroup analysis, it was found that positive SARIFA was associated with poor prognosis in multiple solid tumors. Still, the effect intensity demonstrated certain differences depending on the type of cancer: colorectal cancer (HR = 1.48, 95% CI: 1.37–1.61, I^2^ = 0.0%, *p* = 0.600), gastric cancer/esophageal-gastric cancer (HR = 1.65, 95% CI: 1.44–1.89, I^2^ = 30.9%, *p* = 0.215), pancreatic ductal adenocarcinoma (HR = 1.64, 95% CI: 1.10–2.43), and prostate cancer (HR = 1.29, 95% CI: 0.76–2.20) (Figure 3). Based on geographical regions, subgroup analysis was conducted; the region with the largest number, Germany, was classified as group 1, and the remaining regions, Finland, the Netherlands, Hungary, and the UK, were classified as group 2 (other regions). Comparison of Germany (HR = 1.51, 95% CI: 1.29–1.77) and other regions (HR = 1.52, 95% CI: 1.41–1.64) showed that the HR values and confidence intervals were generally consistent. Based on the available data, it can be concluded that geographic factors did not significantly alter the association between SARIFA and overall cancer survival (Figure 4).

**Figure 5:**
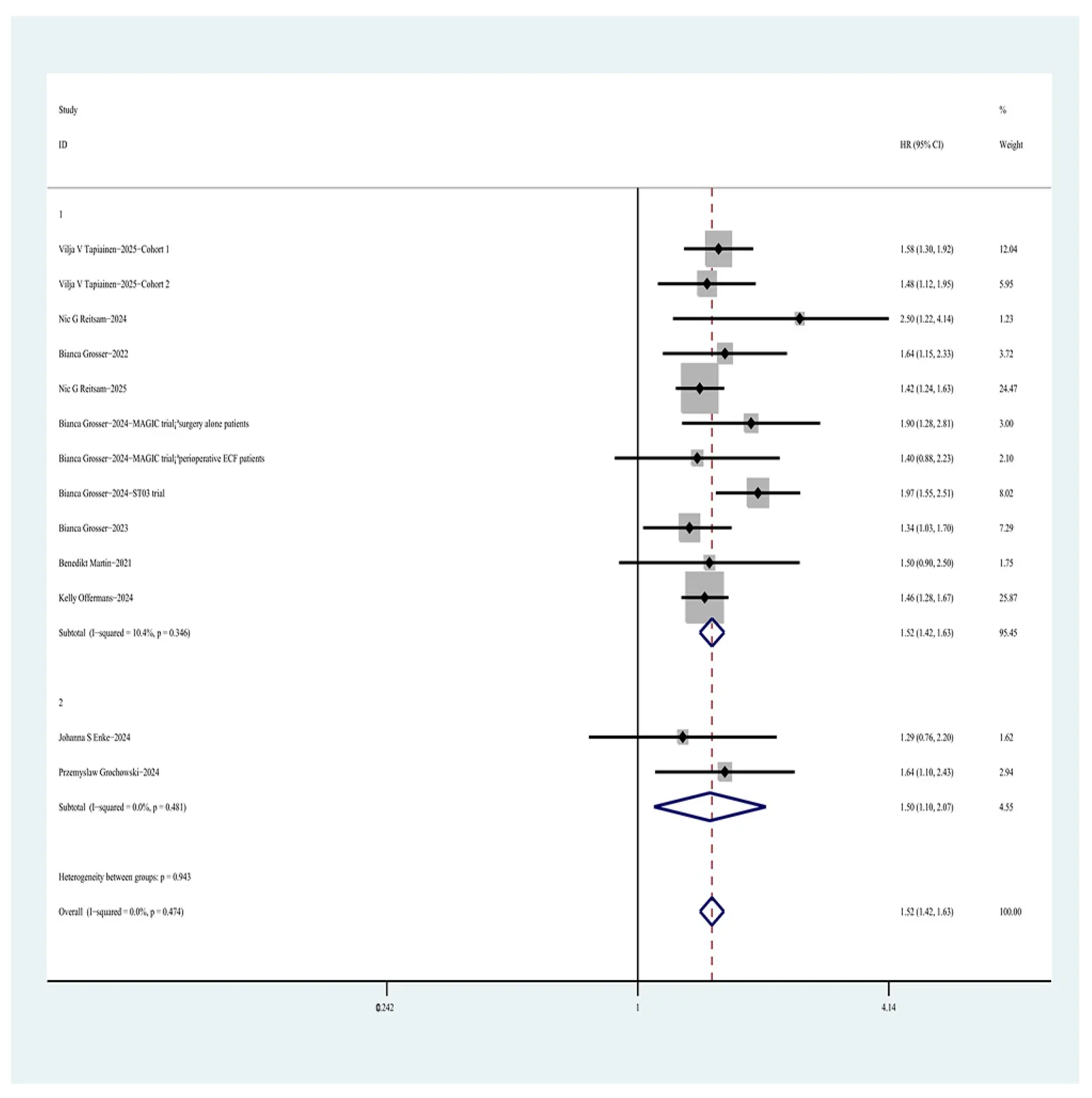
Subgroup analysis results. Subgroup analysis of OS according to the operational definition of SARIFA. Studies using the conventional morphological definition (≥1 tumor gland or ≥5 tumor cell clusters directly contacting adipocytes) are compared with those using modified/other quantitative criteria. The HRs are similar, indicating that definitional variation did not significantly influence the OS estimate [3,6,9,10,12,13,17,18,19,20].

**Figure 6:**
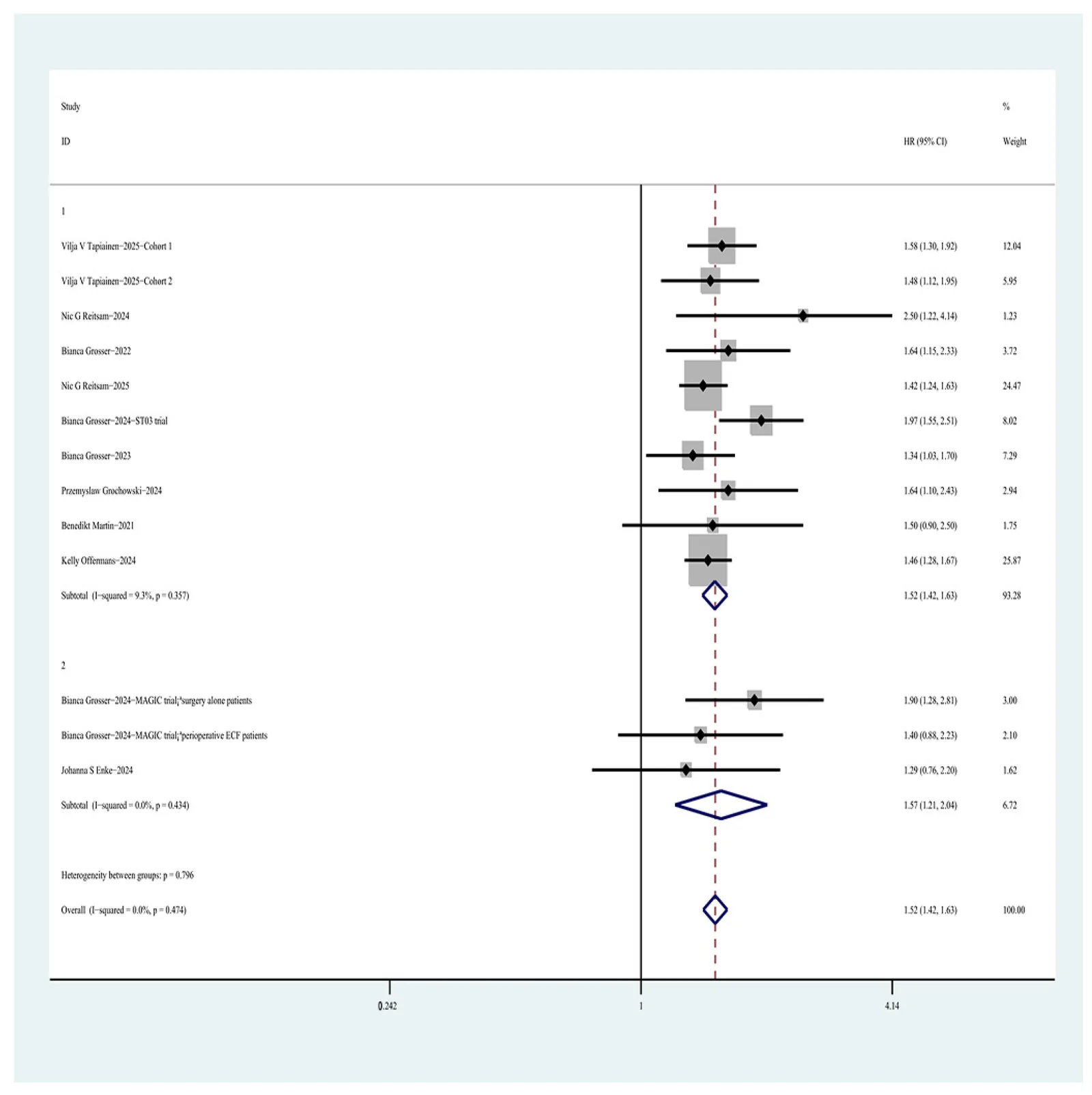
Subgroup analysis results. Subgroup analysis of OS according to statistical adjustment for confounders. Multivariable-adjusted HRs are compared with univariable HRs. The comparable pooled estimates suggest that the OS association is not explained by adjustment for clinicopathological factors [3,6,9,10,12,13,17,18,19,20].

**Figure 7:**
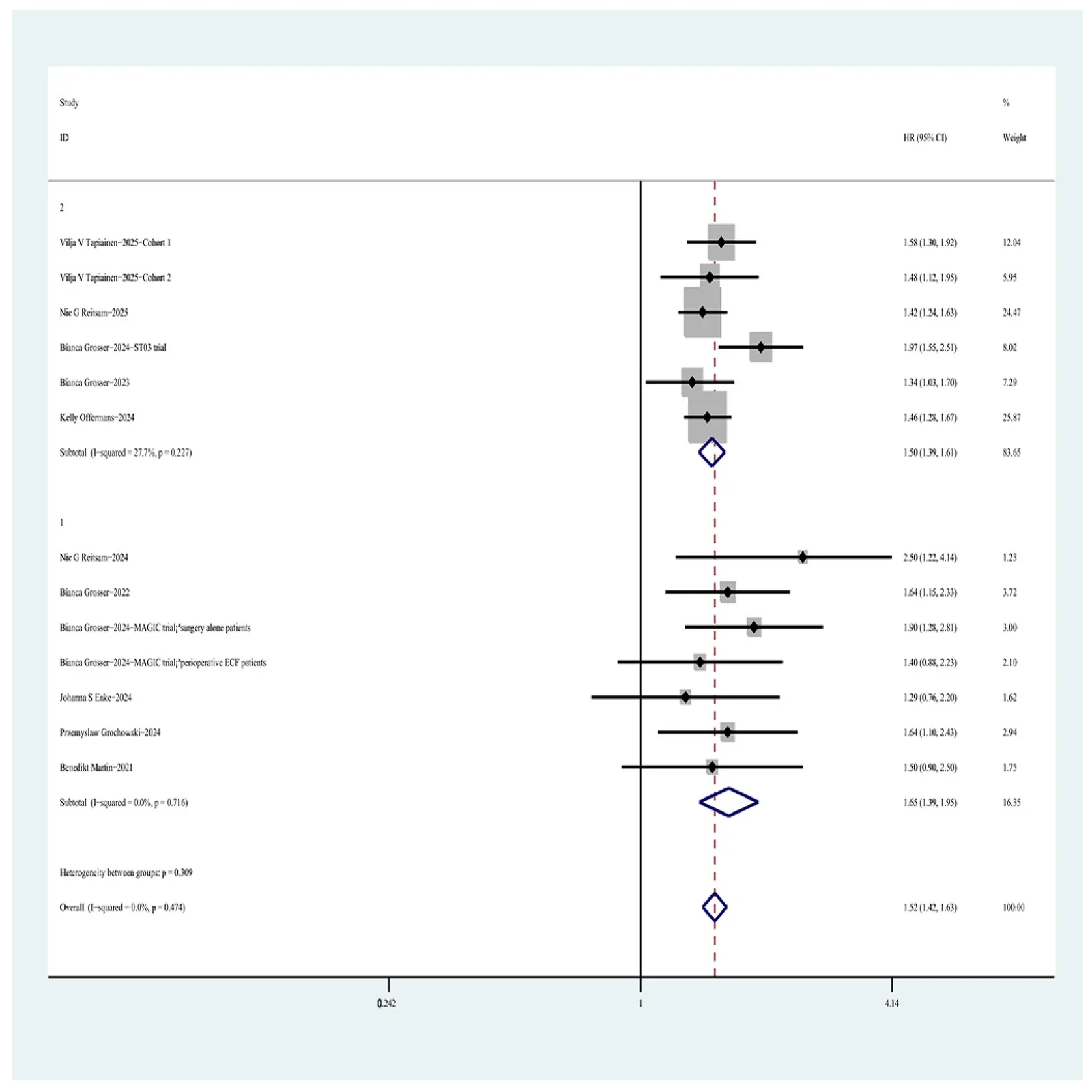
Subgroup analysis results. Subgroup analysis of OS by sample size (small vs. large). The HR estimates are consistent across subgroups [3,6,9,10,12,13,17,18,19,20].

**Figure 8:**
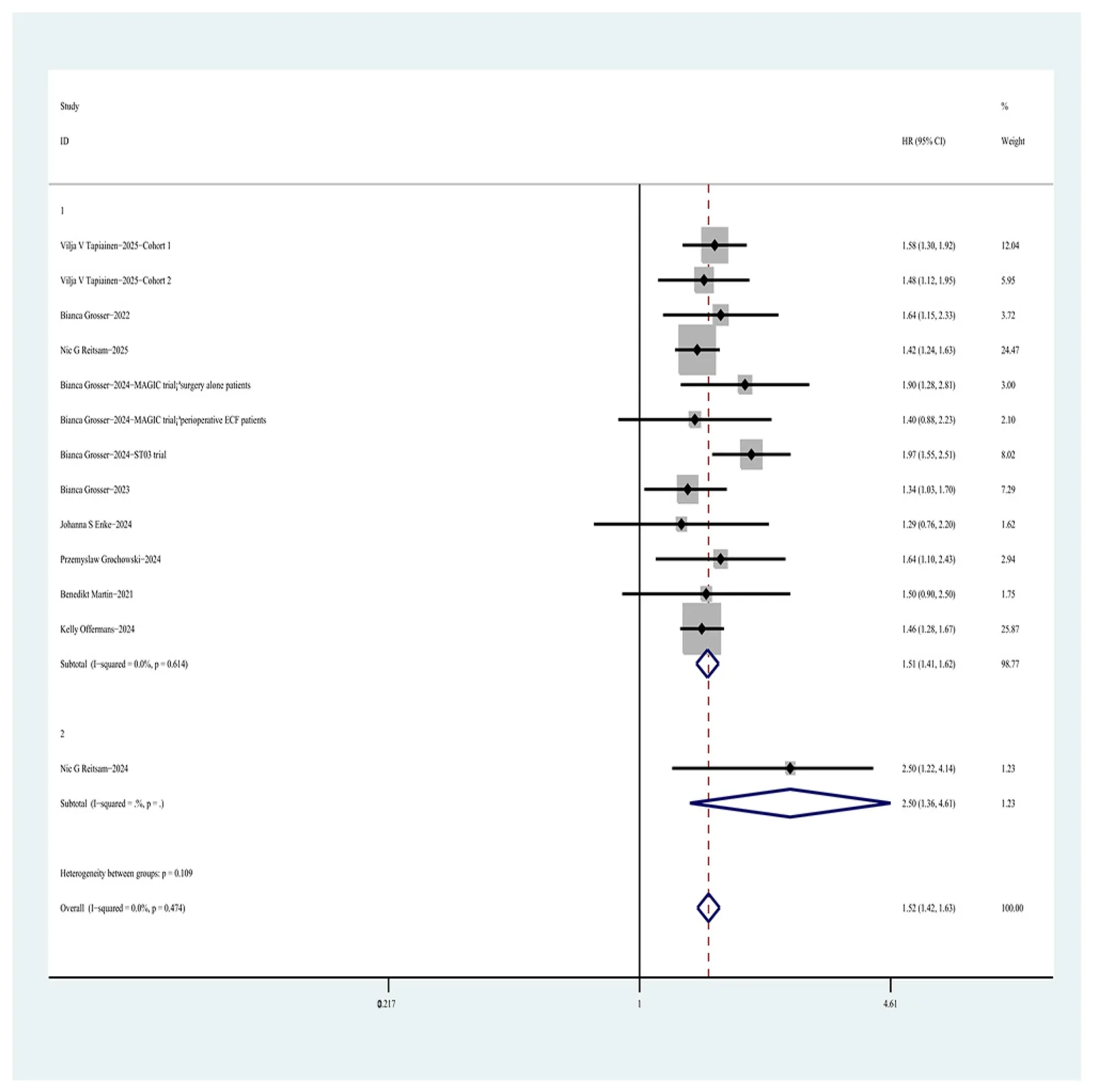
Subgroup analysis results. Subgroup analysis of OS by data source (cohort studies vs. TCGA public data). The HR for TCGA was higher but based on limited data [3,6,9,10,12,13,17,18,19,20].

Subgroup analysis was conducted based on different definitions of SARIFA: the main definition method (HR = 1.52, 95% CI: 1.42–1.63, I^2^ = 10.4%, *p* = 0.346) and other definition methods (HR = 1.50, 95% CI: 1.10–2.07, I^2^ = 0.0%, *p* = 0.481) (Figure 5). The definition-based subgroup analysis was performed because the operational criteria for SARIFA were not fully uniform across tumor types. Most colorectal and gastric/oesophagogastric studies used the conventional morphological definition based on direct tumor–adipocyte contact at the invasive front, whereas pancreatic and prostate studies applied modified or quantitative criteria. This methodological variation could potentially have contributed to between-study differences in effect estimates. To examine whether the prognostic association of SARIFA was influenced by adjustment for established clinicopathological confounders, we performed subgroup analyses according to whether multivariable-adjusted or univariable effect estimates were reported. Results for the subgroup analyzed with multivariate adjustment (HR = 1.52, 95% CI: 1.42–1.63, I^2^ = 9.3%, *p* = 0.357) and the subgroup analyzed with univariate analysis (HR = 1.57, 95% CI: 1.21–2.04, I^2^ = 0.0%, *p* = 0.434) suggested that the observed association between SARIFA positivity and OS was not substantially explained by differences in statistical adjustment (Figure 6). Subgroup analysis was conducted based on sample size, for the subgroup with a small sample size (HR = 1.65, 95% CI: 1.39–1.95, I^2^ = 0.0%, *p* = 0.716) and the subgroup with a large sample size (HR = 1.50, 95% CI: 1.39–1.61, I^2^ = 27.7%, *p* = 0.227) (Figure 7). Subgroup analysis was also performed based on different data sources: free cohorts from various studies (HR = 1.51, 95% CI: 1.41–1.62, I^2^ = 0.0%, *p* = 0.614) and the public data from TCGA (HR = 2.50, 95% CI: 1.36–4.61) (Figure 8). The data indicated no significant difference in the combined HR values across subgroups, and no significant heterogeneity was observed in the analysis.

### 3.3. Sensibility Analysis and Publication Bias

**Figure 9:**
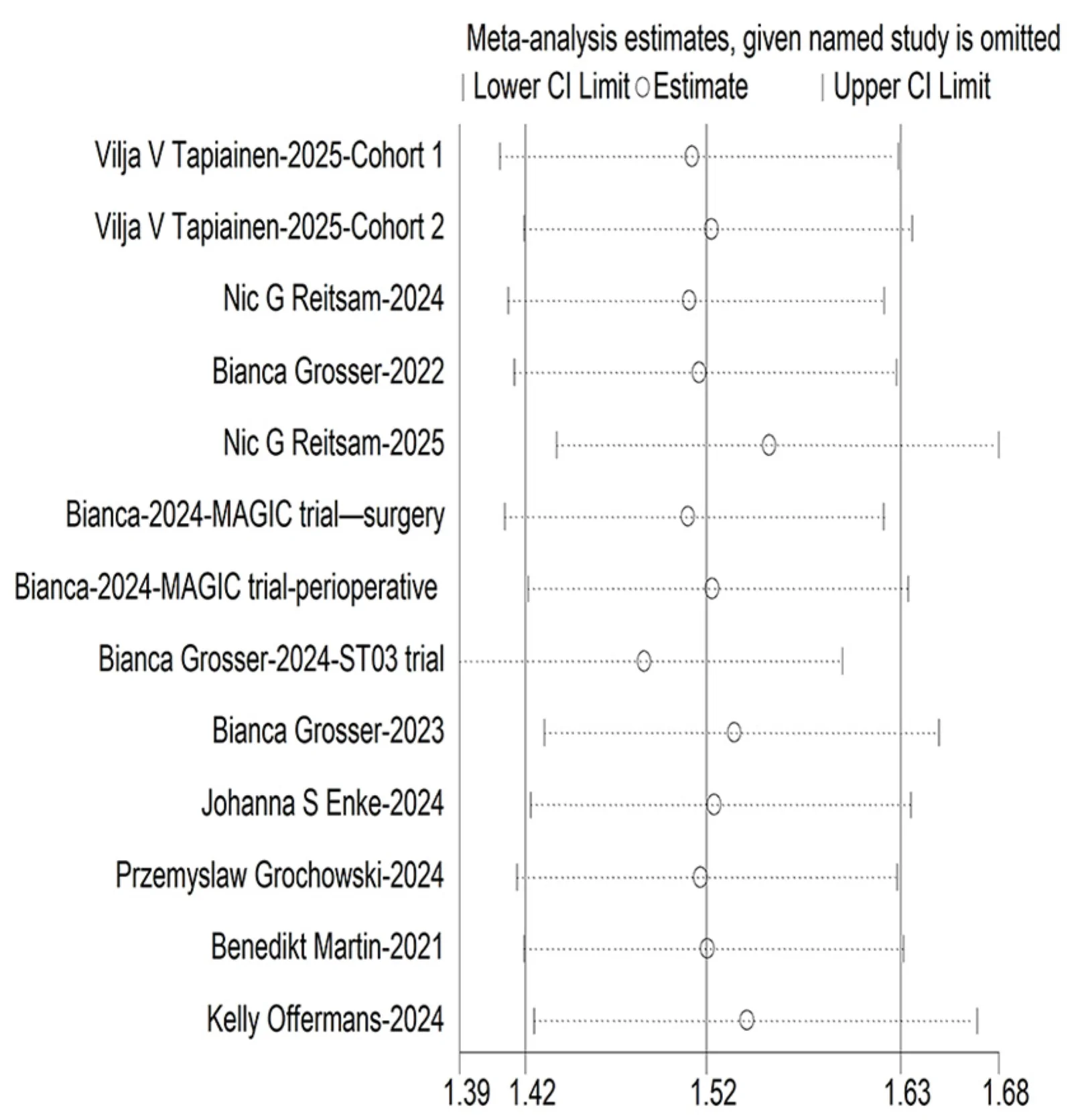
Sensitivity analysis between SARIFA positivity and overall survival (OS) [3,6,9,10,12,13,17,18,19,20].

**Figure 10:**
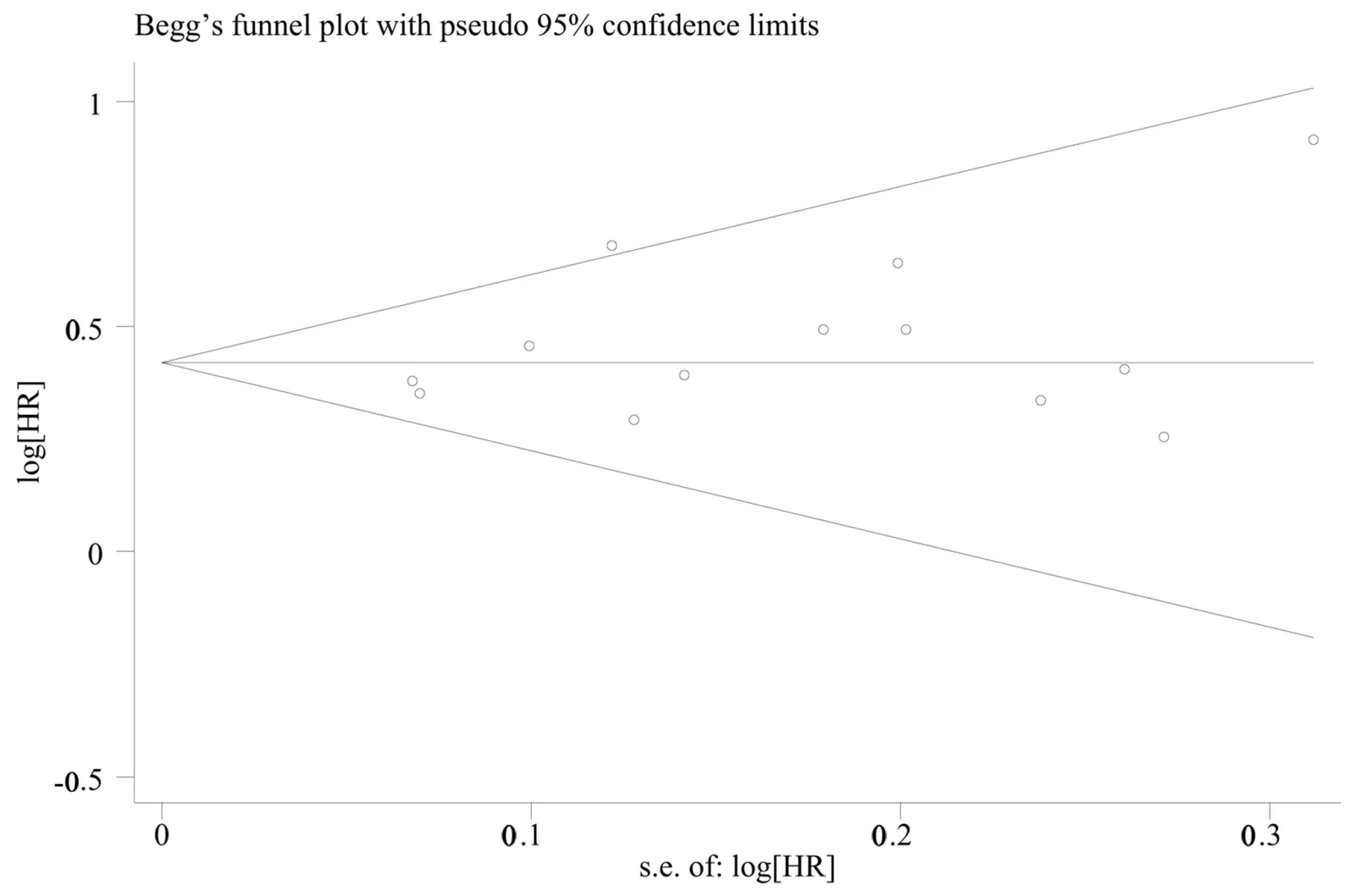
Test results for publication bias. Begg’s funnel plot for publication bias in the OS meta-analysis. Symmetrical distribution suggests no significant bias (Begg’s *p* = 0.393).

**Figure 11:**
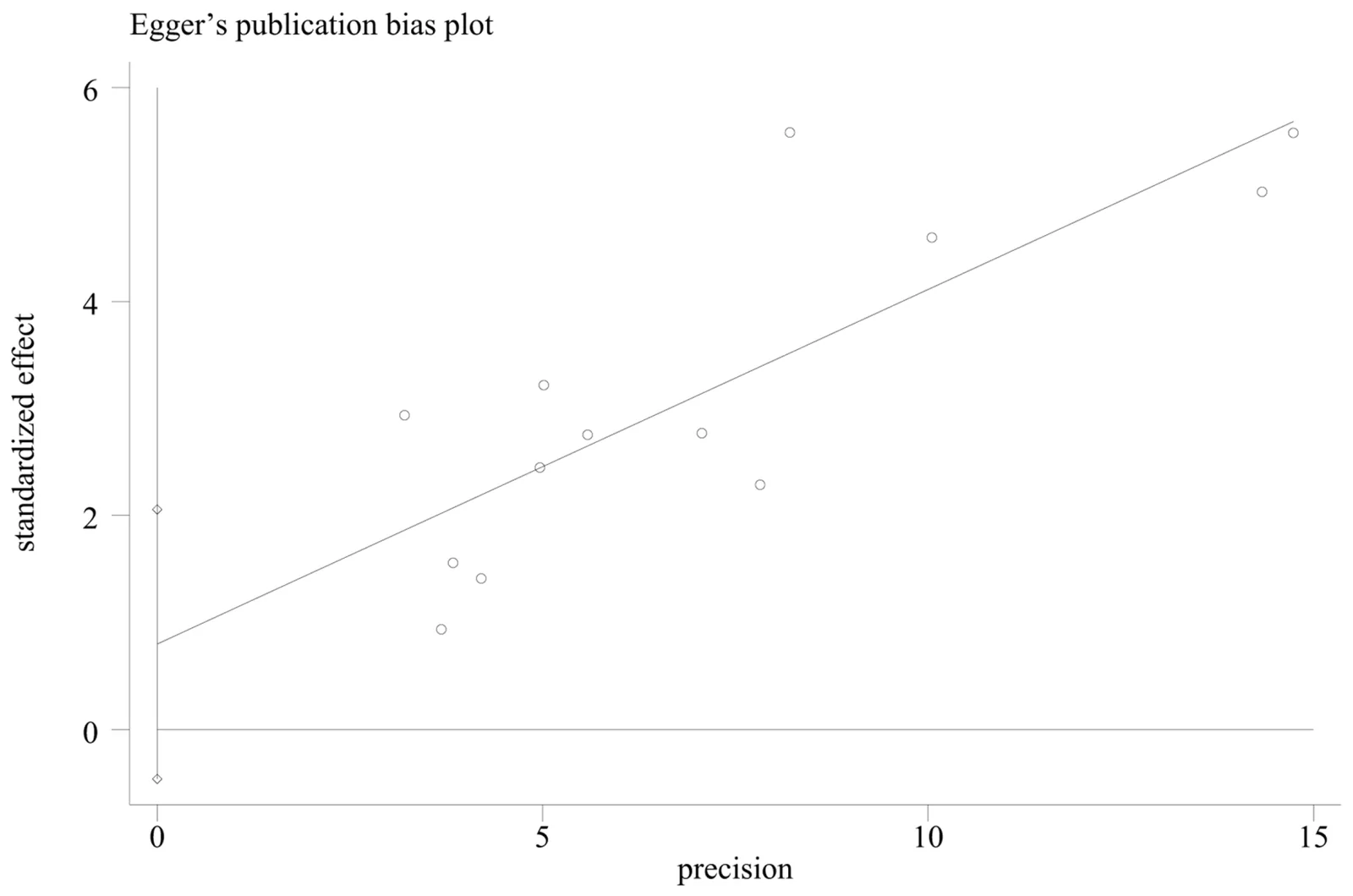
Test results for publication bias. Egger’s test for publication bias in the OS meta-analysis. *p* = 0.189 indicates no significant publication bias.

As shown in Figure 9, after eliminating each study individually, the combined indicators of the remaining studies did not change significantly. This indicates that no single study significantly influenced our main conclusion and that the results were robust. Visual and quantitative assessment of publication bias was conducted using Begg’s and Egger’s funnel plots (Figure 10 and Figure 11). Begg’s test (*p* = 0.393) and Egger’s test (*p* = 0.189) both indicated that there was no statistically significant publication bias in this analysis.

### 3.4. Certainty of Evidence

The certainty of evidence for the association between SARIFA positivity and each outcome was assessed using the GRADE approach. The certainty of evidence for overall survival was considered moderate, whereas the certainty of evidence for clinicopathological outcomes varied because of substantial heterogeneity, imprecision, and potential publication bias. The detailed GRADE assessment is presented in Supplementary Table S3.

**Figure 12:**
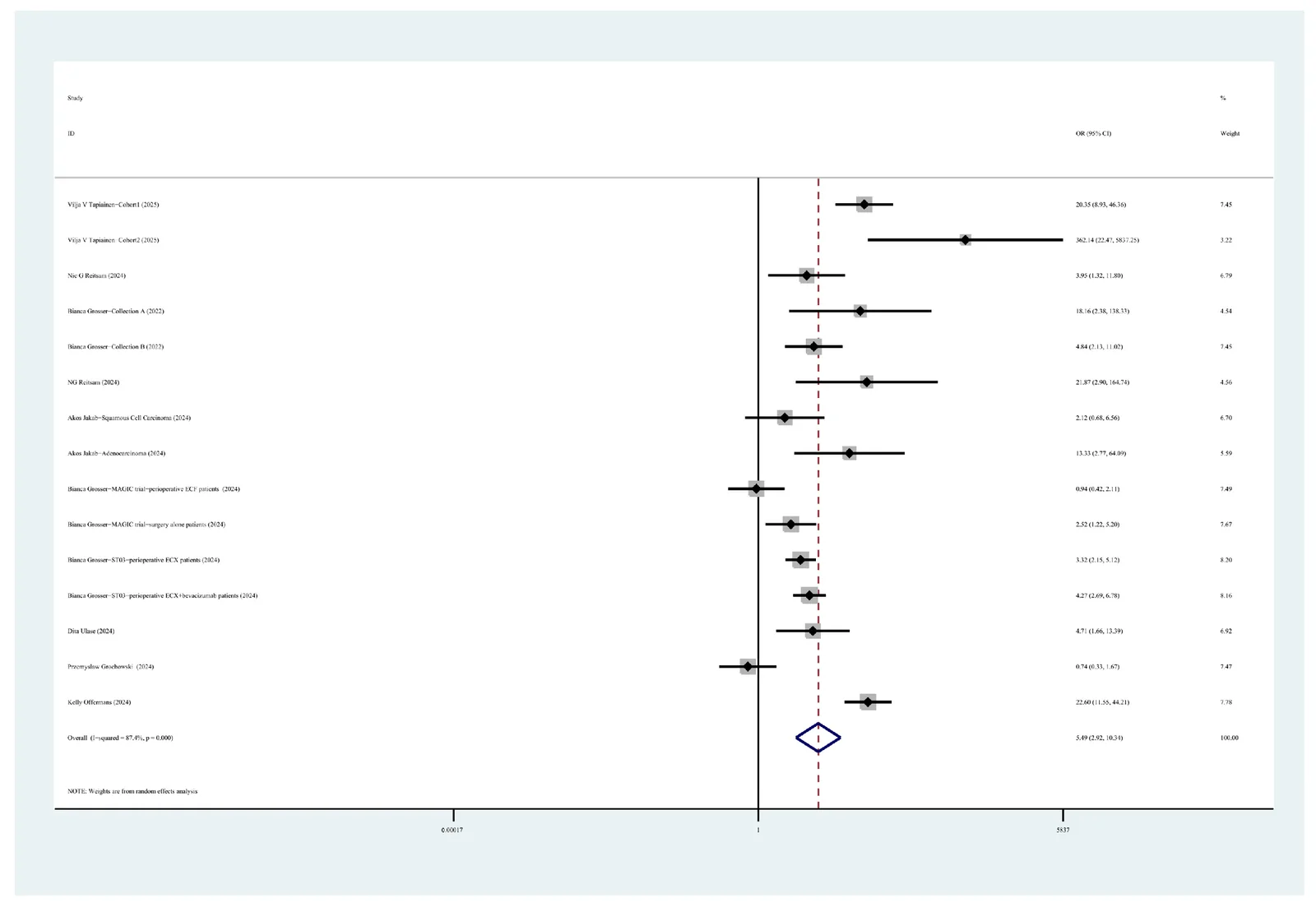
Forest plot of SARIFA positivity and clinical stage. Forest plot of the association between SARIFA positivity and advanced pT stage (T1–2 vs. T3–4). Pooled OR = 5.49 with substantial heterogeneity (I^2^ = 87.4%) [6,8,9,12,17,18,19,22,23].

**Figure 13:**
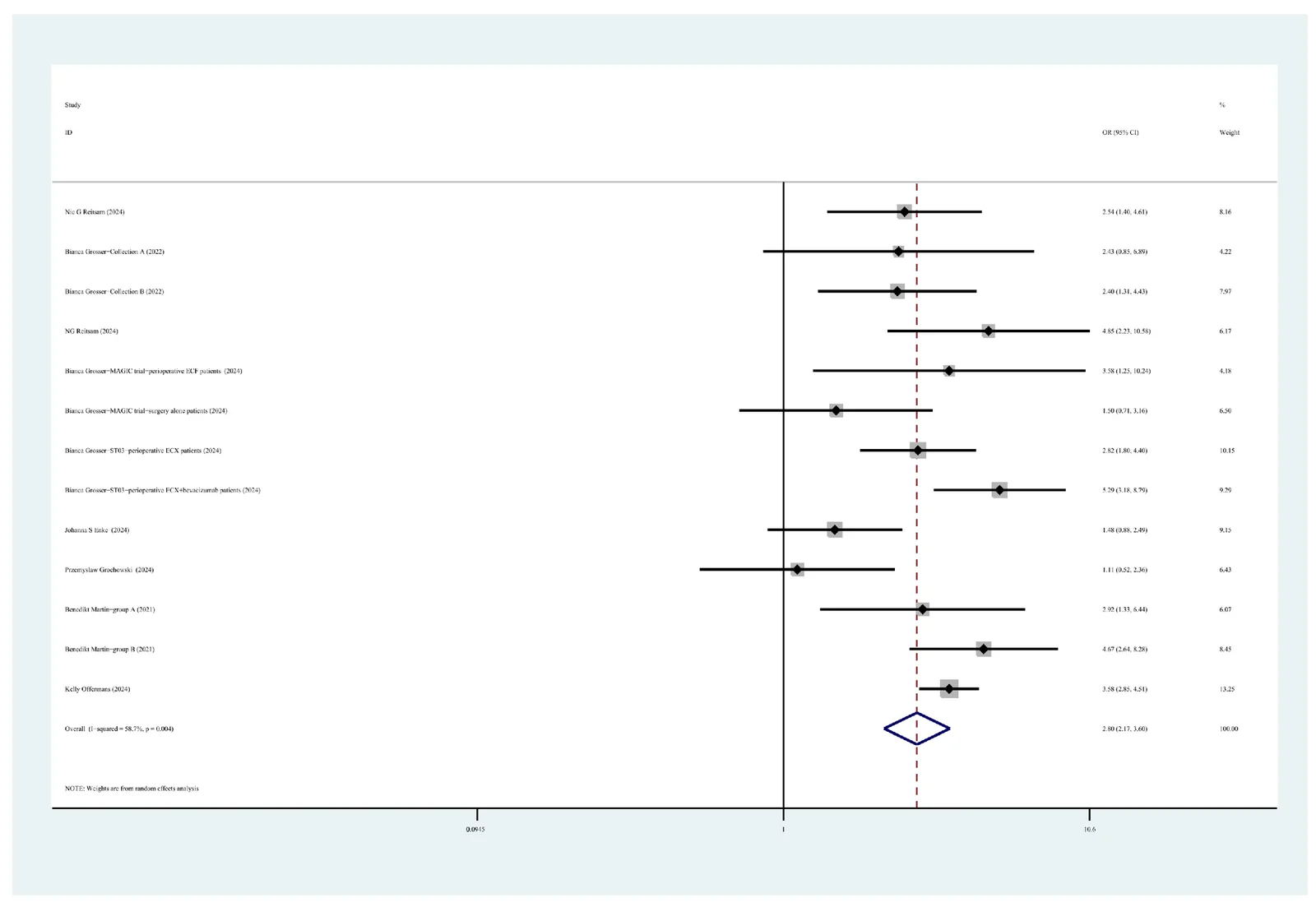
Forest plot of SARIFA positivity and clinical stage. Forest plot for advanced pN stage (N0 vs. N1+). Pooled OR = 2.80, I^2^ = 58.7% [3,6,8,9,10,17,18,19].

**Figure 14:**
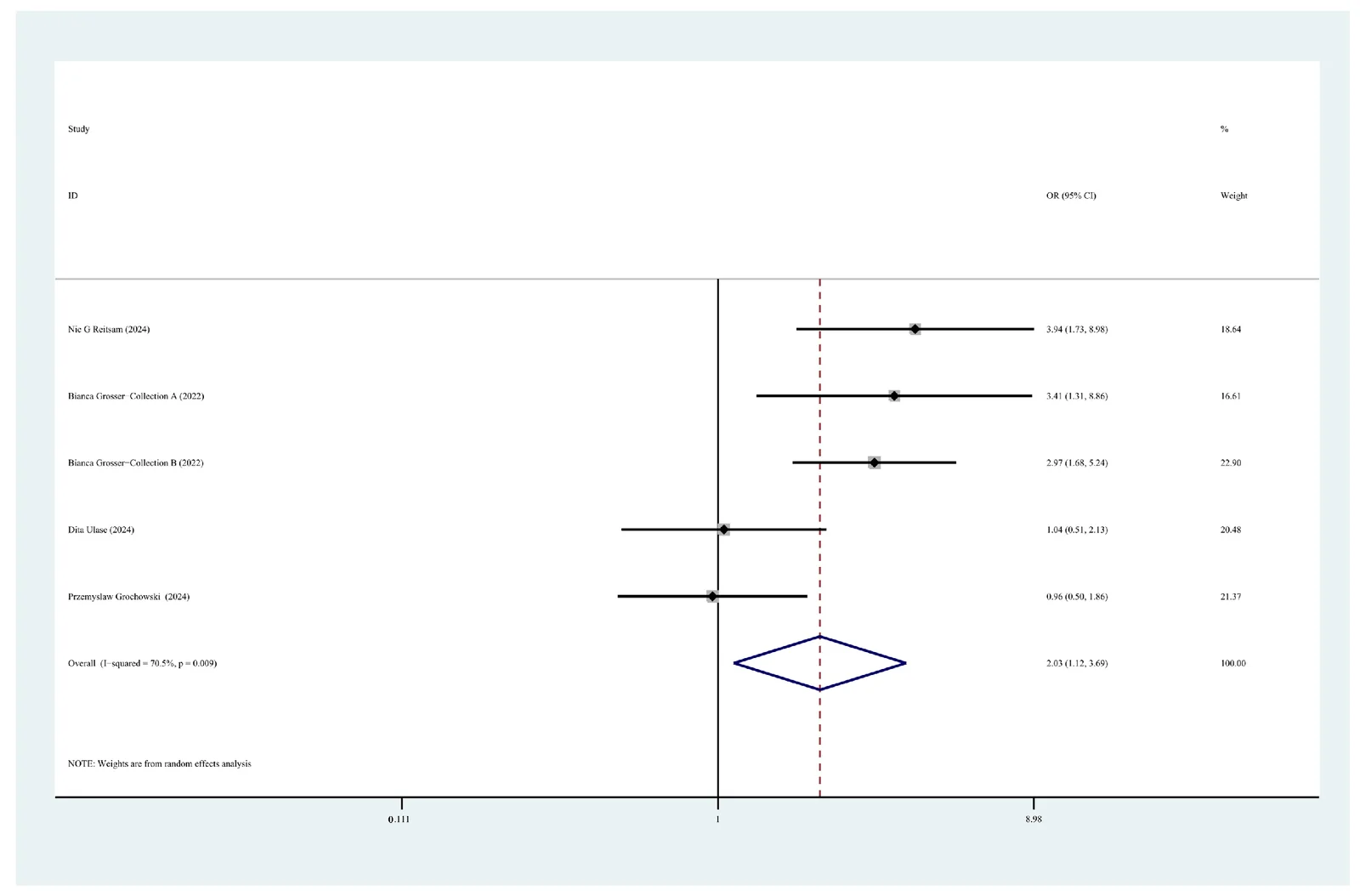
Forest plot of SARIFA positivity and clinical stage. Forest plot for pM1 status (M0 vs. M1). Pooled OR = 2.03, I^2^ = 70.5% [6,9,17,23].

**Figure 15:**
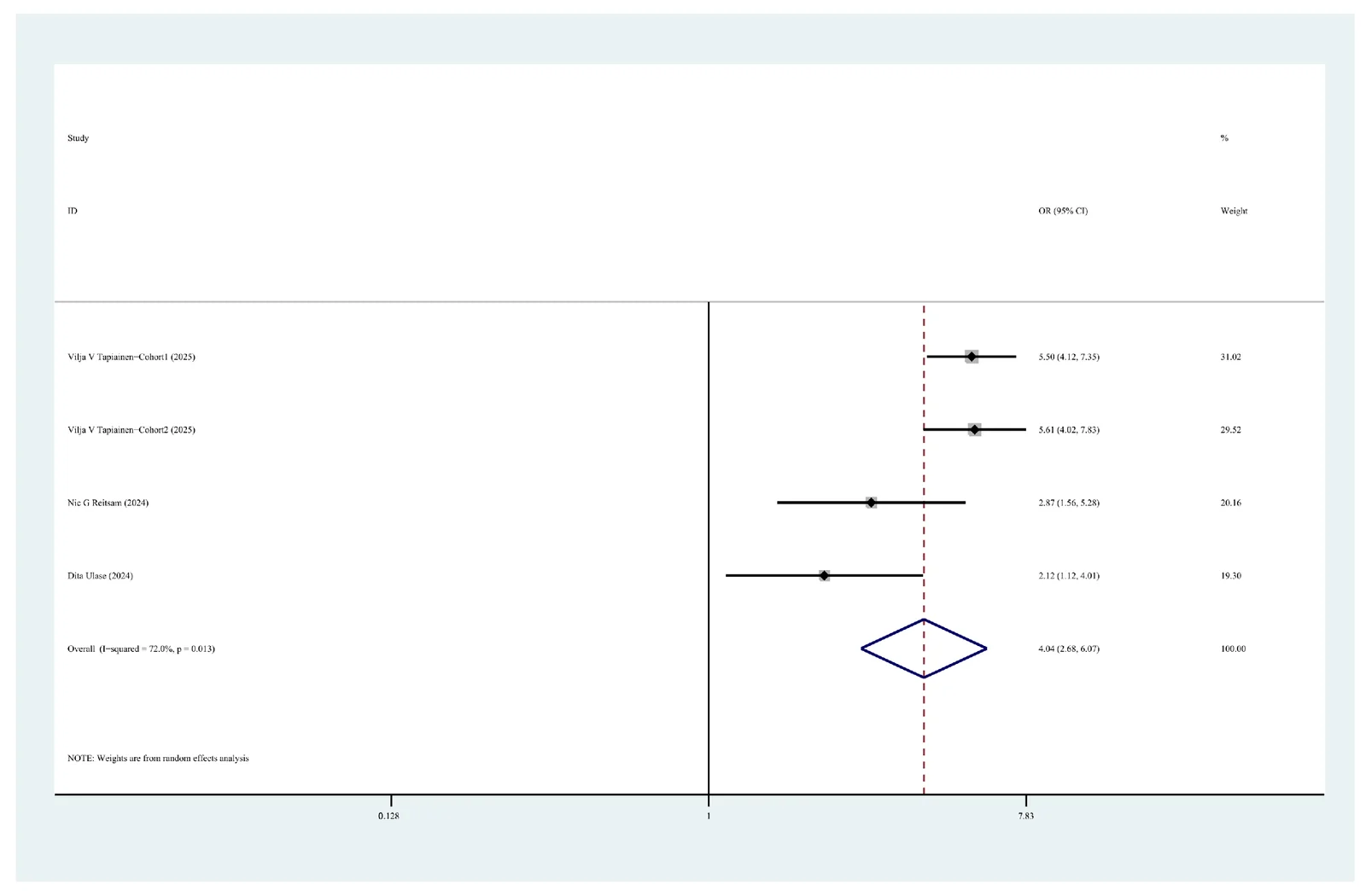
Forest plot of SARIFA positivity and clinical stage. Forest plot for advanced AJCC stage (I–II vs. III–IV). Pooled OR = 4.04, I^2^ = 72.0% [12,17,23].

**Figure 16:**
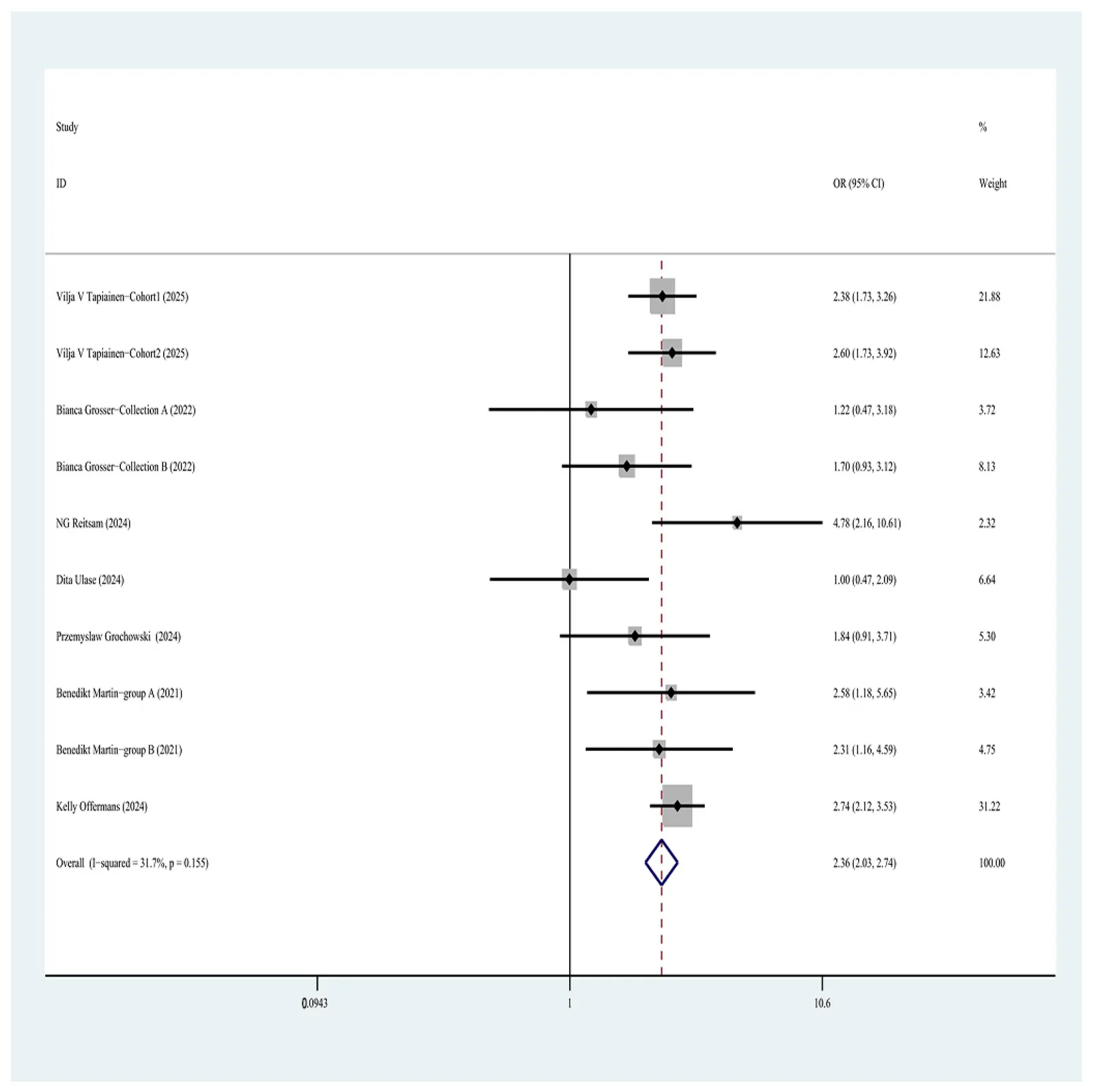
Forest plot of SARIFA positivity and clinical stage. Forest plot for high pathological grade (low vs. high grade). Pooled OR = 2.36, I^2^ = 31.7% [3,6,8,9,12,18,23].

With regard to the relationship between SARIFA positivity and clinical stage, there were 11 studies researching SARIFA positivity and the occurrence of high-grade tumors, involving 6847 patients and 18 cohorts. Different studies used different grading methods for tumors. In pT staging, T1/2 was defined as low-grade tumors, and T3/4 as high-grade tumors; the analysis result showed that the relationship between SARIFA positivity and the occurrence of high-grade tumors was OR = 5.49, 95% CI: 2.92–10.34, I^2^ = 87.4%, *p* = 0.0 (Figure 12). In pN staging, N0 was defined as low-grade tumors, and N1 and above as high-grade tumors; the analysis result was that the relationship between SARIFA positivity and the occurrence of high-grade tumors was OR = 2.80, 95% CI: 2.17–3.60, I^2^ = 58.7%, *p* = 0.004 (Figure 13). In pM staging, M0 was defined as low-grade tumors, and M1 and above as high-grade tumors; the analysis result was that the relationship between SARIFA positivity and the occurrence of high-grade tumors was OR = 2.03, 95% CI: 1.12–3.69, I^2^ = 70.5%, *p* = 0.009 (Figure 14). In AJCC staging, stage 1/2 was defined as low-grade tumors, and stage 3/4 as high-grade tumors; the analysis result was that the relationship between SARIFA positivity and the occurrence of high-grade tumors was OR = 4.04, 95% CI: 2.68–6.07, I^2^ = 72.0%, *p* = 0.013 (Figure 15). The analysis result of tumor pathological grading showed that the relationship between SARIFA positivity and the occurrence of high-grade tumors was OR = 2.36, 95% CI: 2.03–2.74, I^2^ = 31.7%, *p* = 0.155 (Figure 16). Apart from tumor pathological grading, results from other tumor staging methods also showed significant heterogeneity.

**Figure 17:**
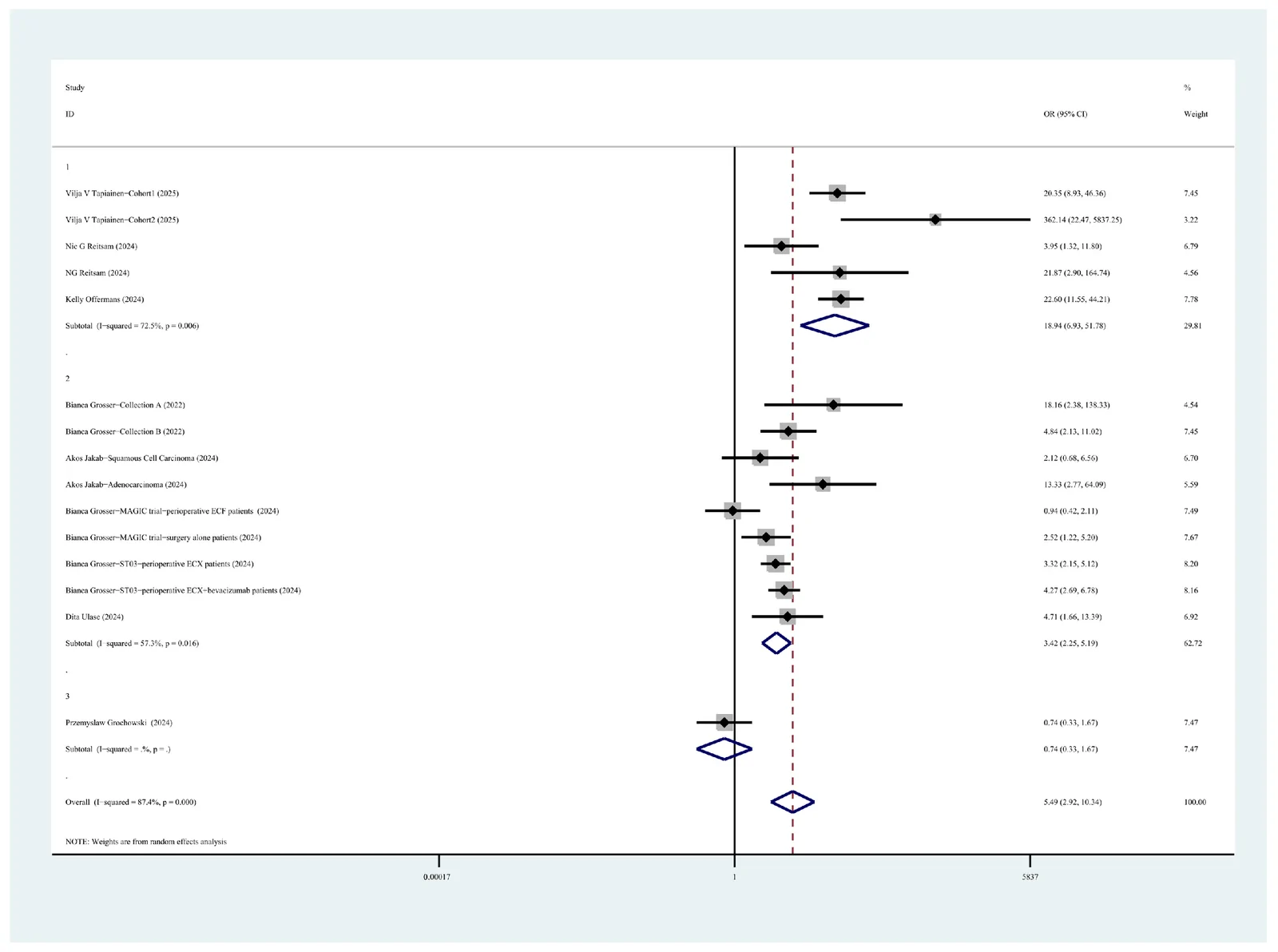
Subgroup analysis of clinical stage. Subgroup analysis of pT stage by cancer type. Colorectal cancer shows the strongest association, while pancreatic cancer (single study) shows no significant effect [6,8,9,12,17,18,19,22,23].

**Figure 18:**
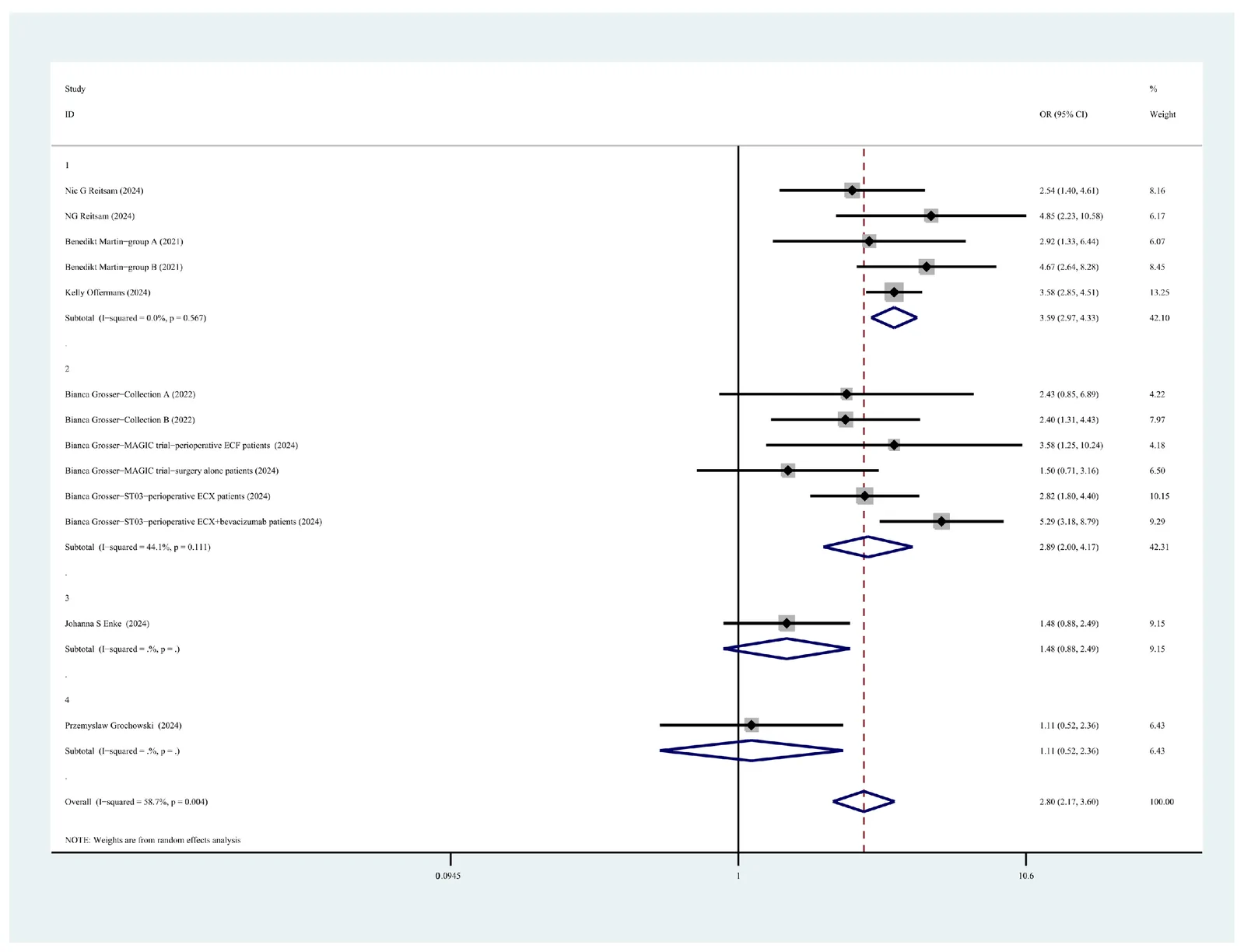
Subgroup analysis of clinical stage. Subgroup analysis of pN stage by cancer type [3,6,8,9,10,17,18,19].

**Figure 19:**
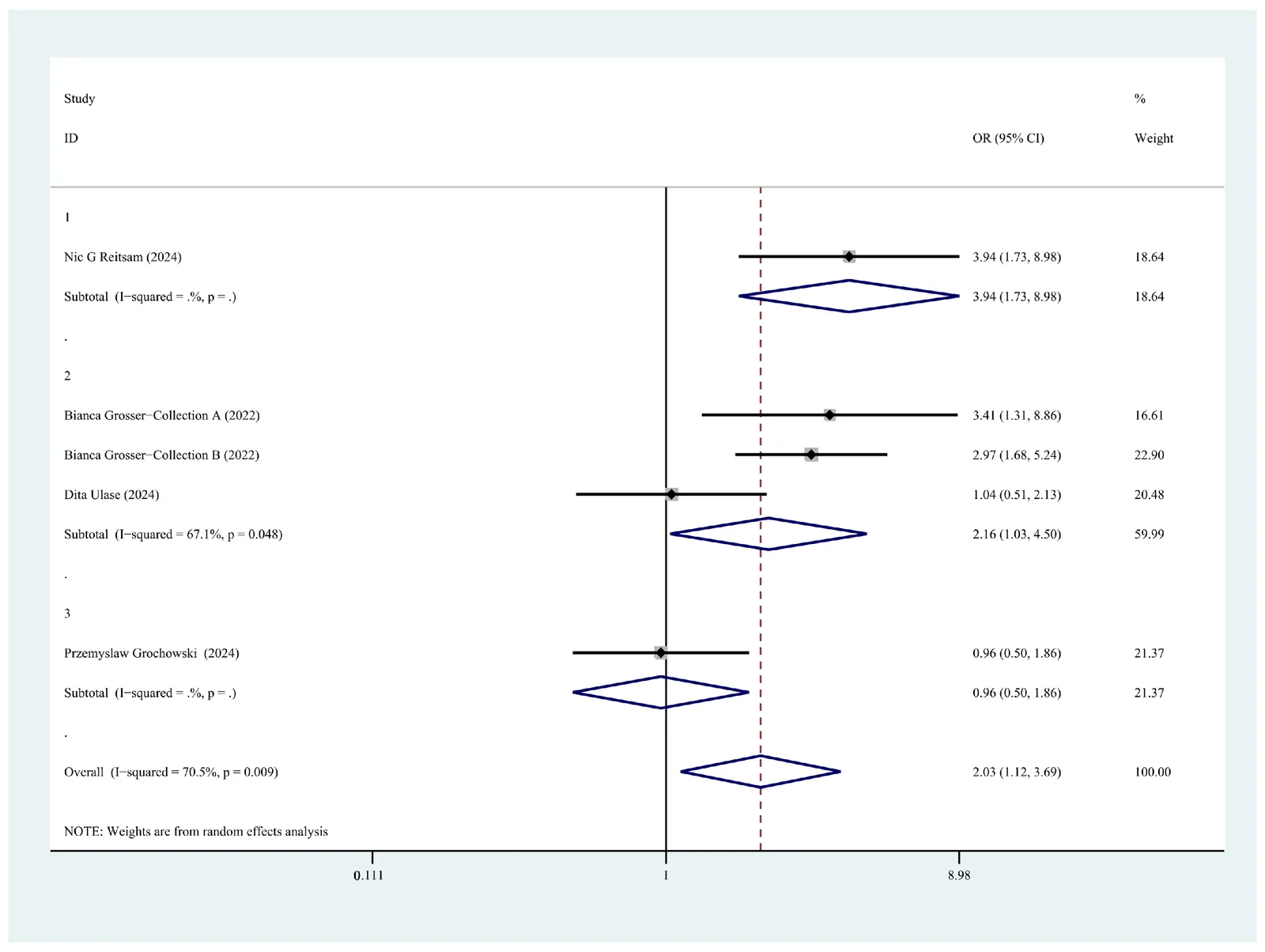
Subgroup analysis of clinical stage. Subgroup analysis of pM status by cancer type [6,9,17,23].

**Figure 20:**
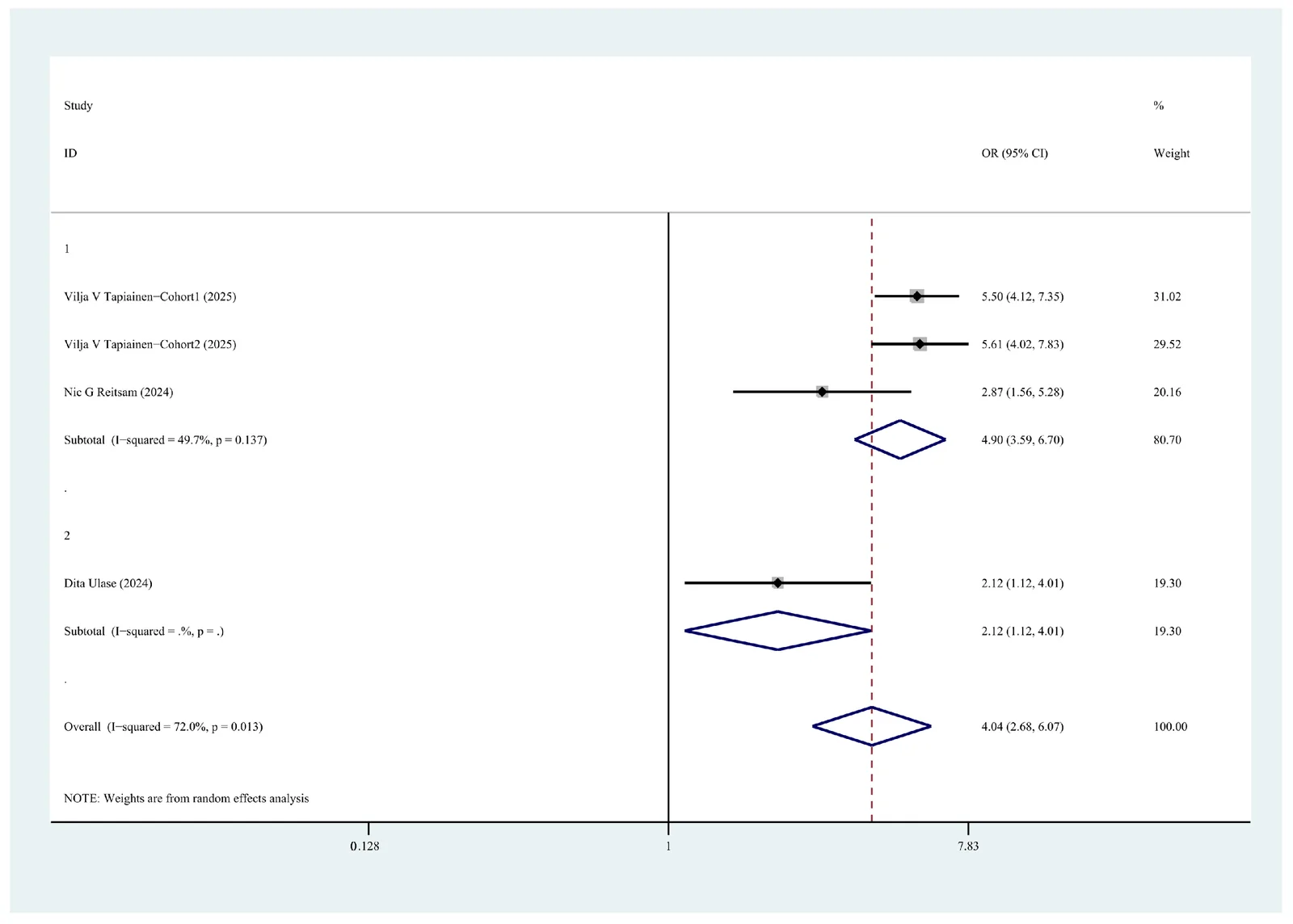
Subgroup analysis of clinical stage. Subgroup analysis of AJCC stage by cancer type [12,17,23].

**Figure 21:**
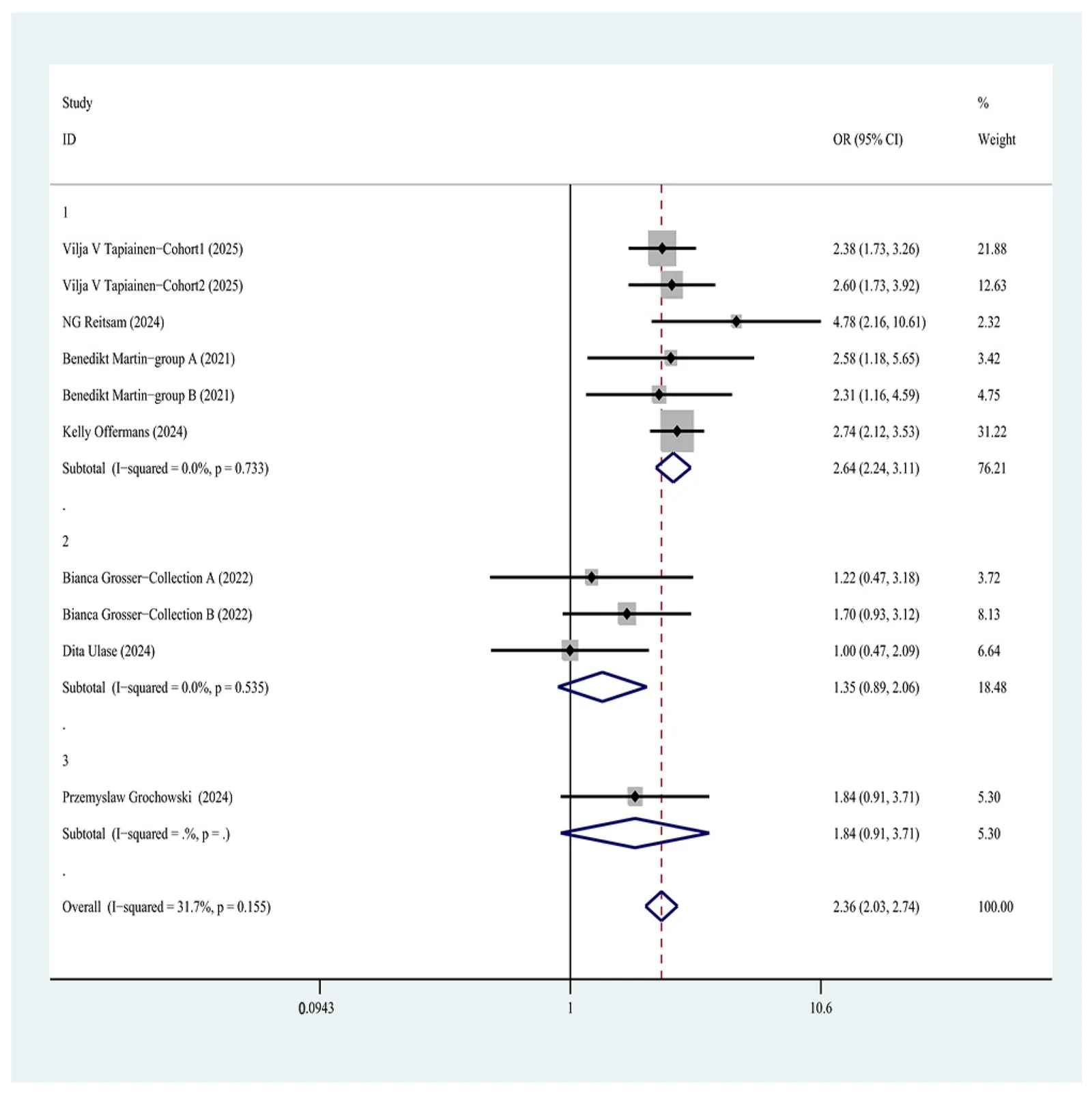
Subgroup analysis of clinical stage. Subgroup analysis of pathological grade by cancer type [3,6,8,9,12,18,23].

To explore sources of heterogeneity, subgroup analyses were conducted by cancer type. pT stage: colorectal cancer (OR = 18.94, 95% CI: 6.93–51.78, I^2^ = 72.5%, *p* = 0.006), gastric cancer/esophageal–gastric cancer (OR = 3.42, 95% CI: 2.25–5.19, I^2^ = 57.3%, *p* = 0.016), pancreatic ductal adenocarcinoma (OR = 0.74, 95% CI: 0.33–1.67, only one study) (Figure 17). pN stage: colorectal cancer (OR = 3.59, 95% CI: 2.97–4.33, I^2^ = 0.0%, *p* = 0.567), gastric cancer/esophageal–gastric cancer (OR = 2.89, 95% CI: 2.00–4.17, I^2^ = 44.1%, *p* = 0.111), prostate cancer (OR = 1.48, 95% CI: 0.88–2.49, only one study), pancreatic ductal adenocarcinoma (OR = 1.11, 95% CI: 0.52–2.36, only one study) (Figure 18). pM stage: colorectal cancer (OR = 3.94, 95% CI: 1.73–8.98, only one study), gastric cancer/esophageal–gastric cancer (OR = 2.16, 95% CI: 1.03–4.50, I^2^ = 67.1%, *p* = 0.048), pancreatic ductal adenocarcinoma (OR = 0.96, 95% CI: 0.50–1.86, only one study) (Figure 19). AJCC stage: colorectal cancer (OR = 4.90, 95% CI: 3.59–6.70, I^2^ = 49.7%, *p* = 0.137), gastric cancer/esophageal–gastric cancer (OR = 2.12, 95% CI: 1.12–4.01, only one study) (Figure 20). Tumor pathological grade: colorectal cancer (OR = 2.64, 95% CI: 2.24–3.11, I^2^ = 0.0%, *p* = 0.733), gastric cancer/esophageal–gastric cancer (OR = 1.35, 95% CI: 0.89–2.06, I^2^ = 0.0%, *p* = 0.535), pancreatic ductal adenocarcinoma (OR = 1.84, 95% CI: 0.91–3.71) (only one study) (Figure 21). No significant heterogeneity was observed in tumor pathological grade and pN stage, while the results for other tumor staging methods showed significant heterogeneity between subgroups.

**Figure 22:**
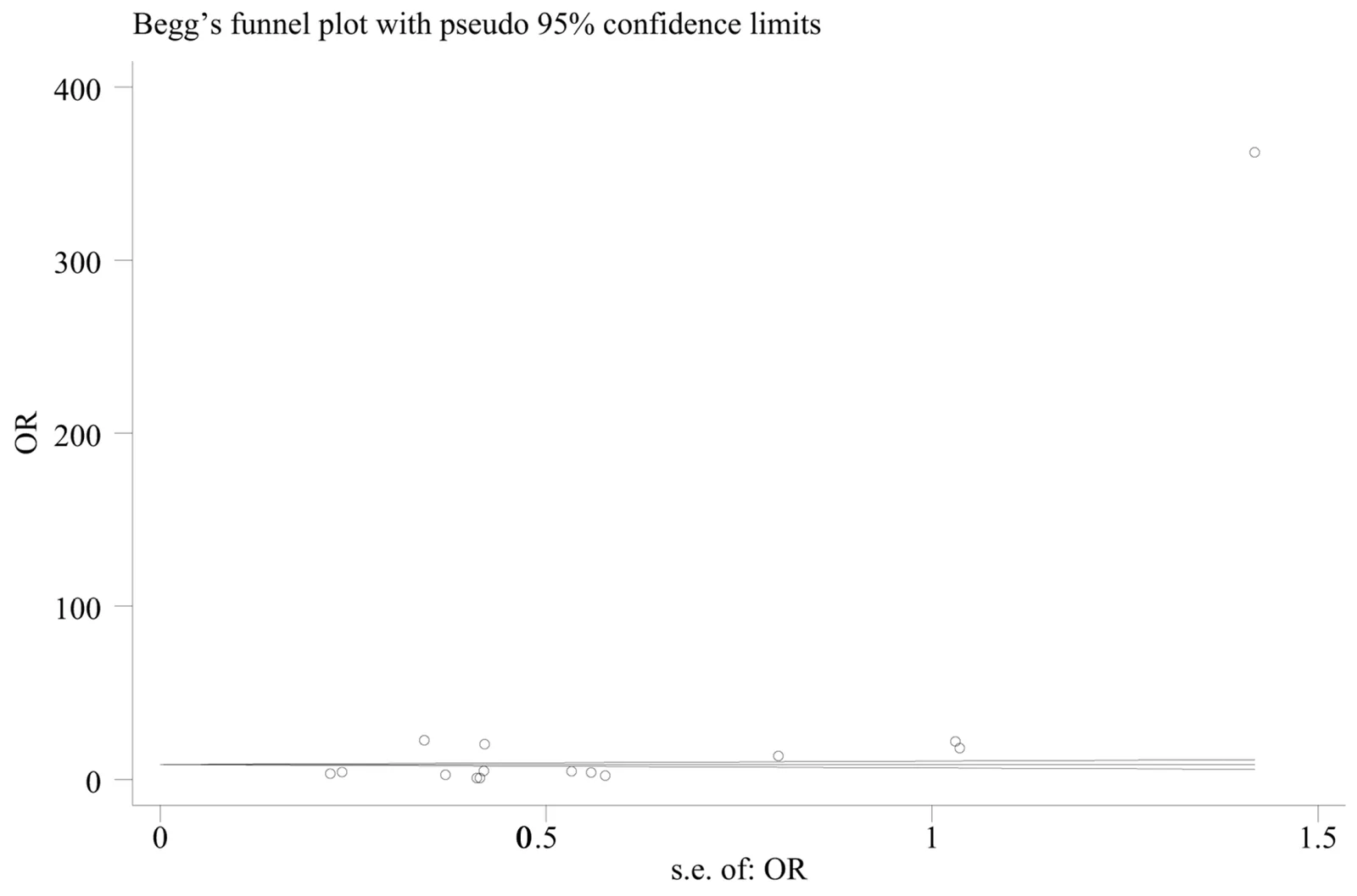
Publication bias at the clinical stage. Begg’s funnel plot for pT stage publication bias (*p* = 0.008).

**Figure 23:**
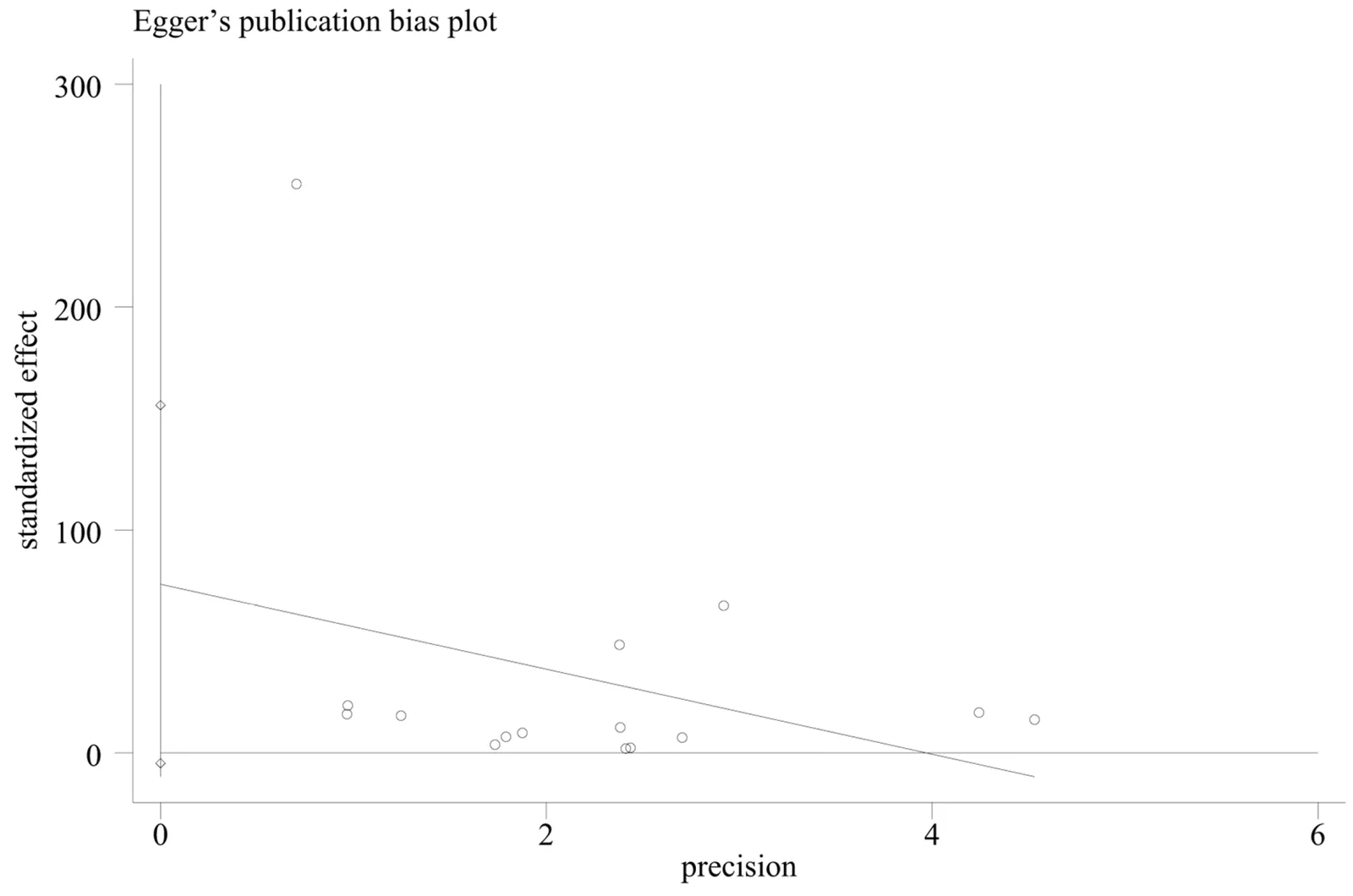
Publication bias at the clinical stage. Egger’s test for pT stage (*p* = 0.063).

**Figure 24:**
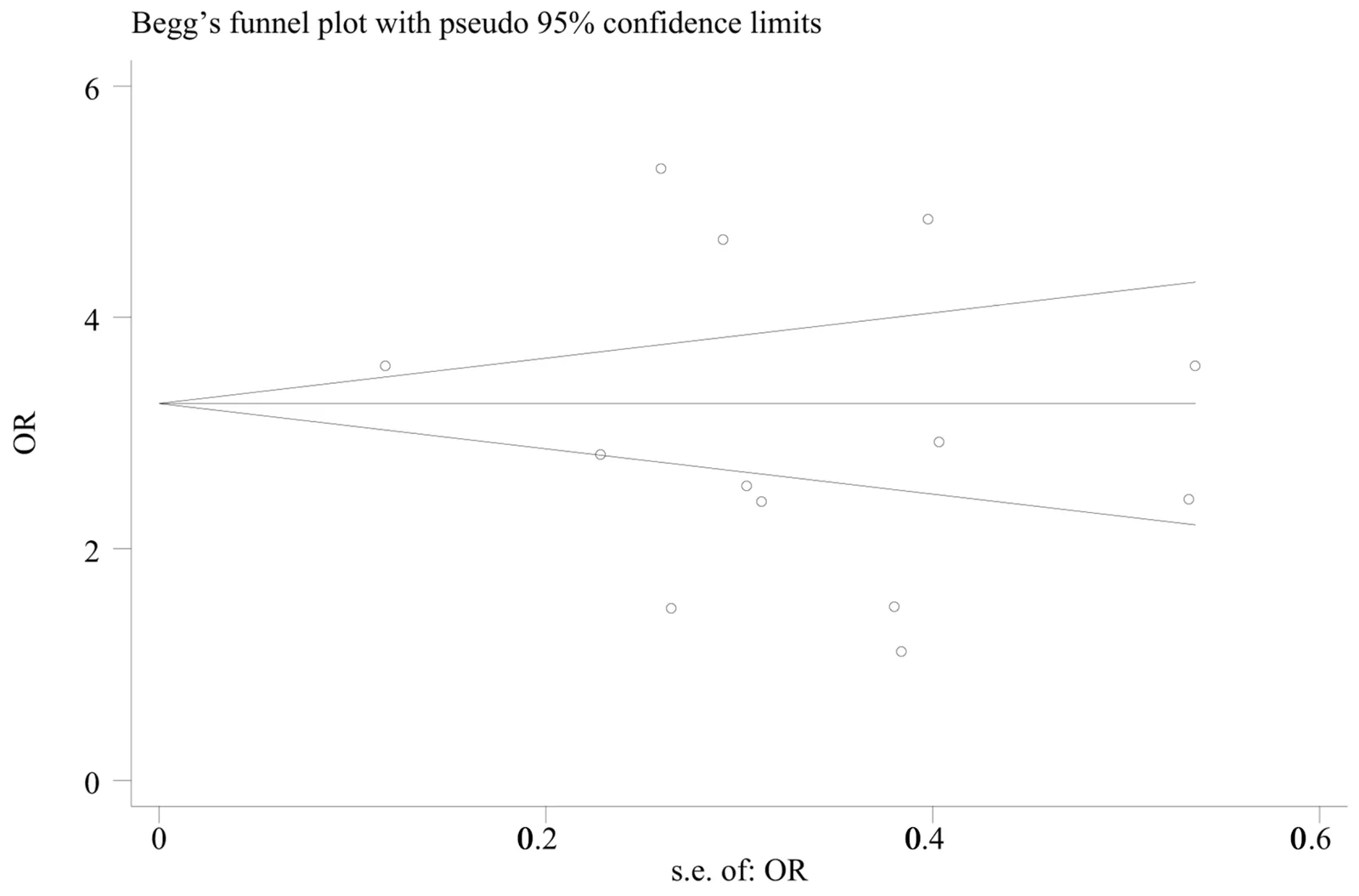
Publication bias at the clinical stage. Begg’s test for pN stage (*p* = 0.760).

**Figure 25:**
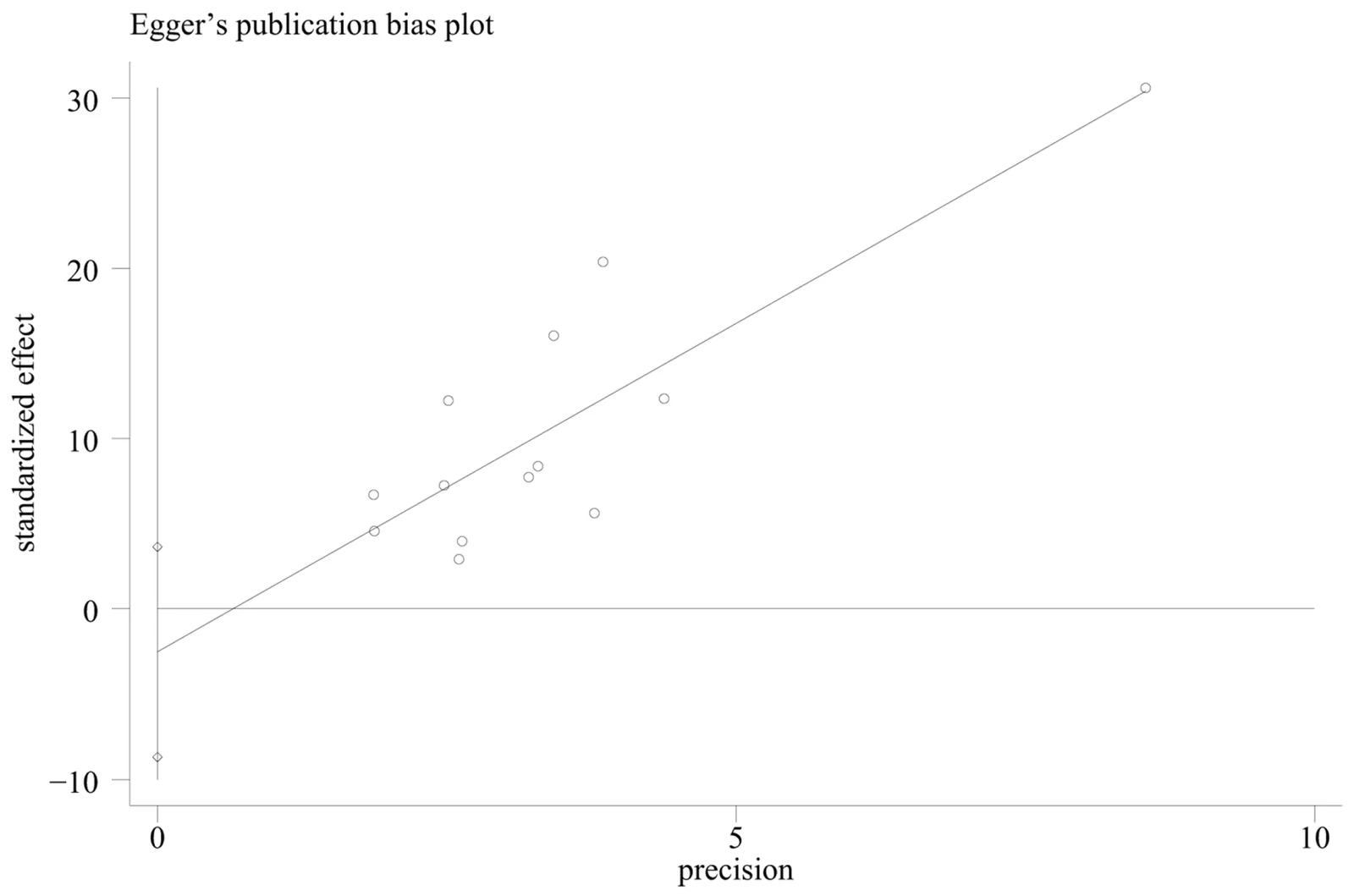
Publication bias at the clinical stage. Egger’s test for pN stage (*p* = 0.386).

**Figure 26:**
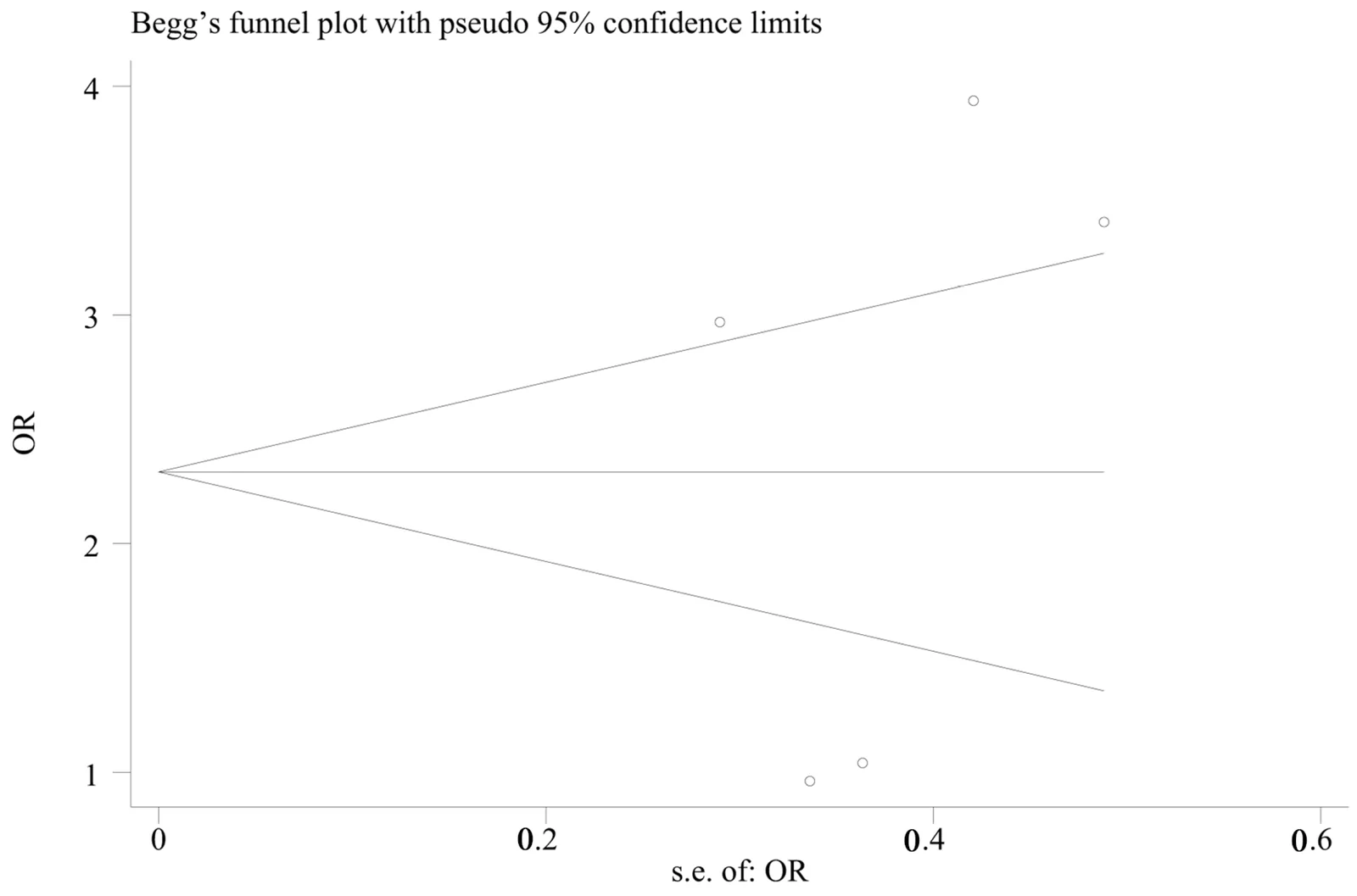
Publication bias at the clinical stage. Begg’s test for pM stage (*p* = 0.806).

**Figure 27:**
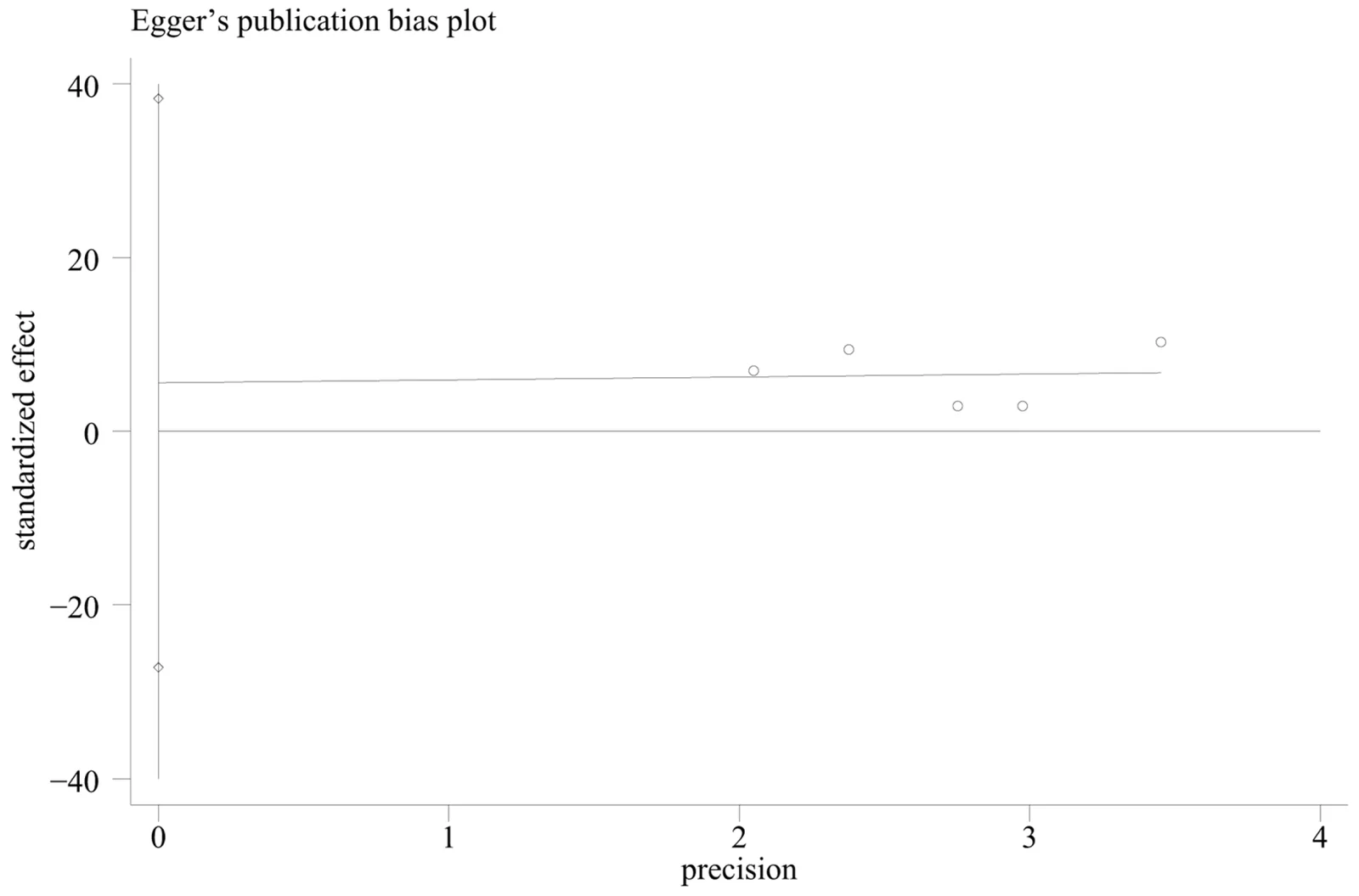
Publication bias at the clinical stage. Egger’s test for pM stage (*p* = 0.628).

**Figure 28:**
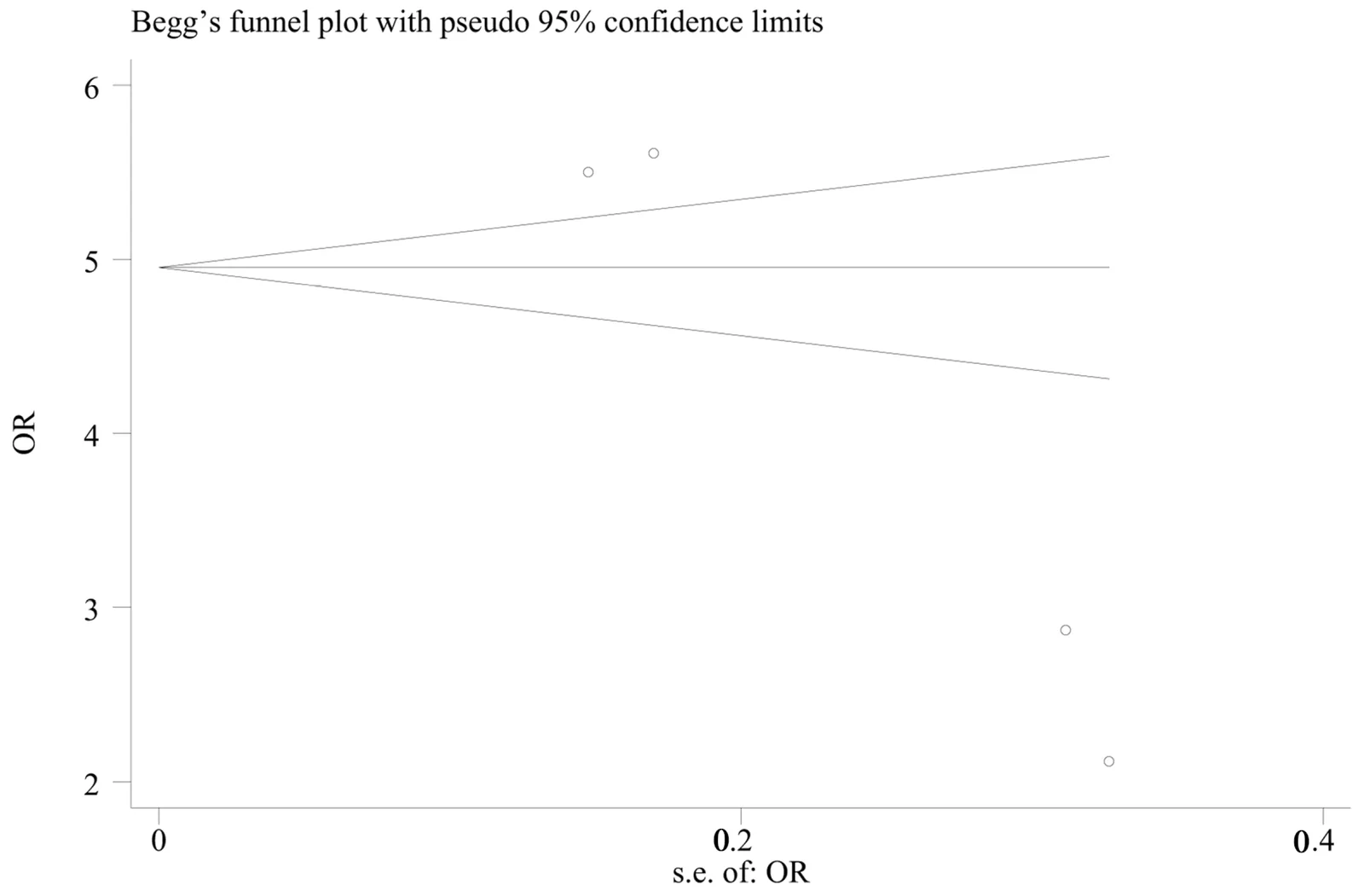
Publication bias at the clinical stage. Begg’s test for AJCC stage (*p* = 0.089).

**Figure 29:**
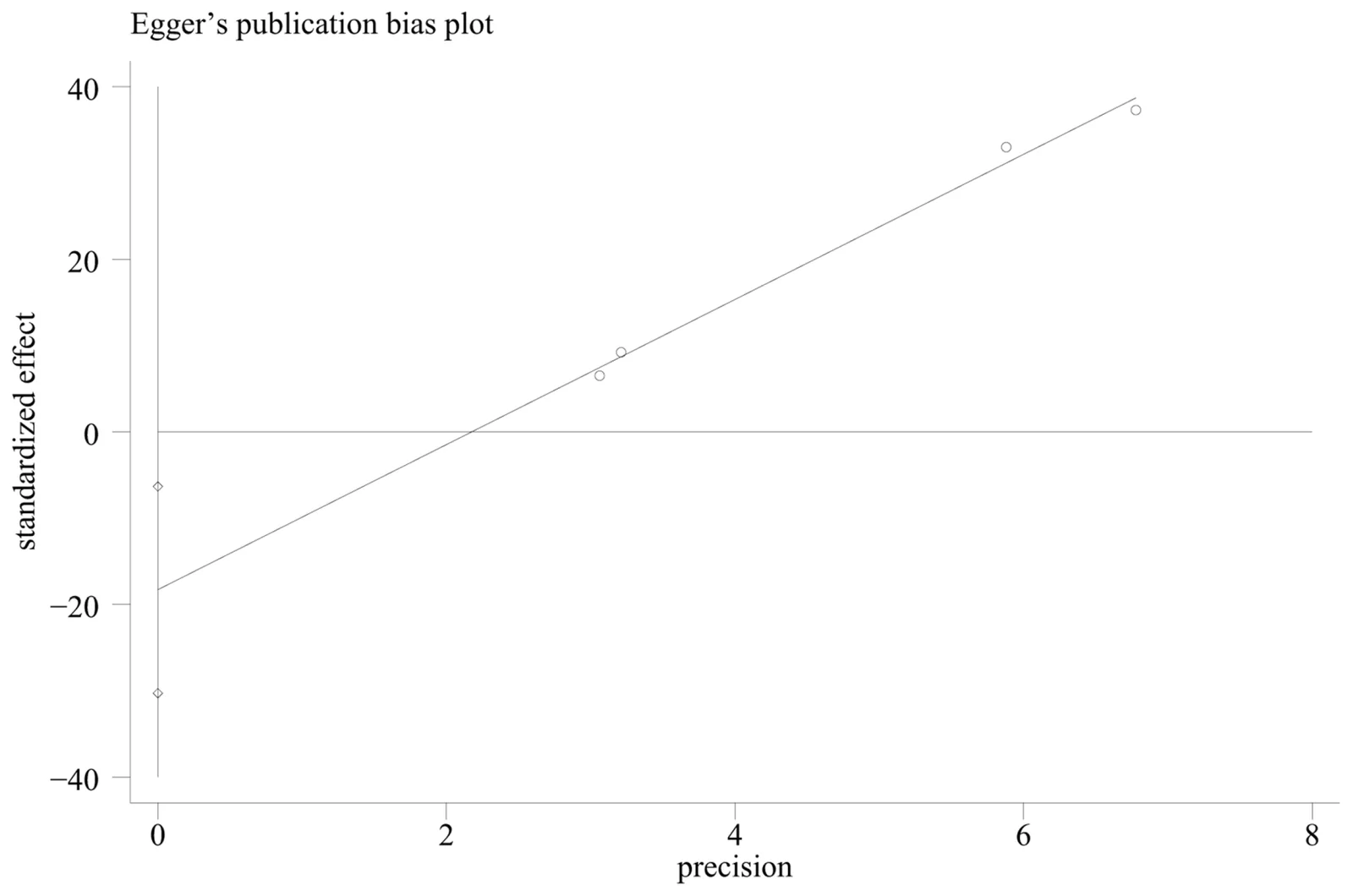
Publication bias at the clinical stage. Egger’s test for AJCC stage (*p* = 0.022).

**Figure 30:**
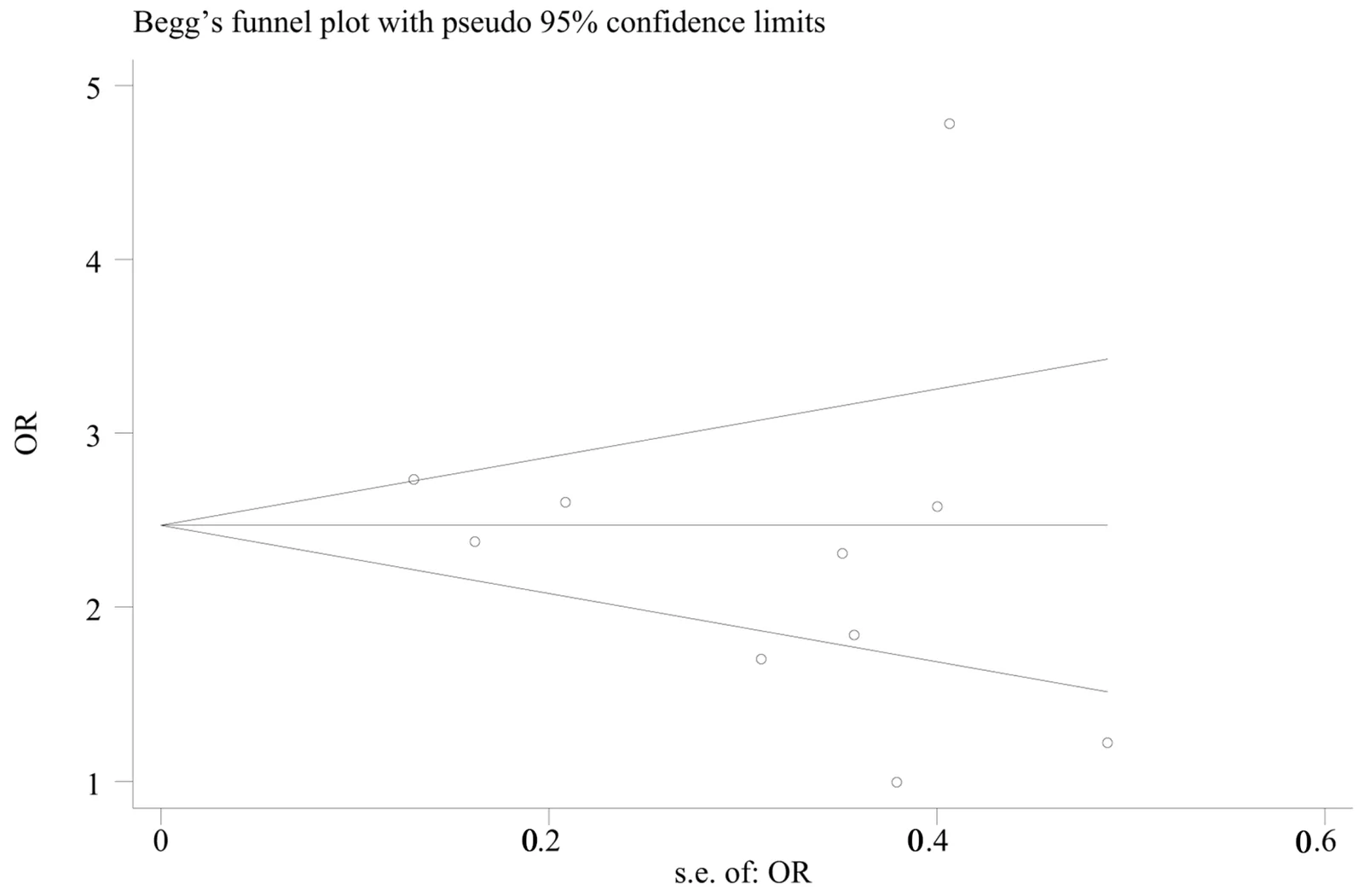
Publication bias at the clinical stage. Begg’s test for pathological grade (*p* = 0.474).

**Figure 31:**
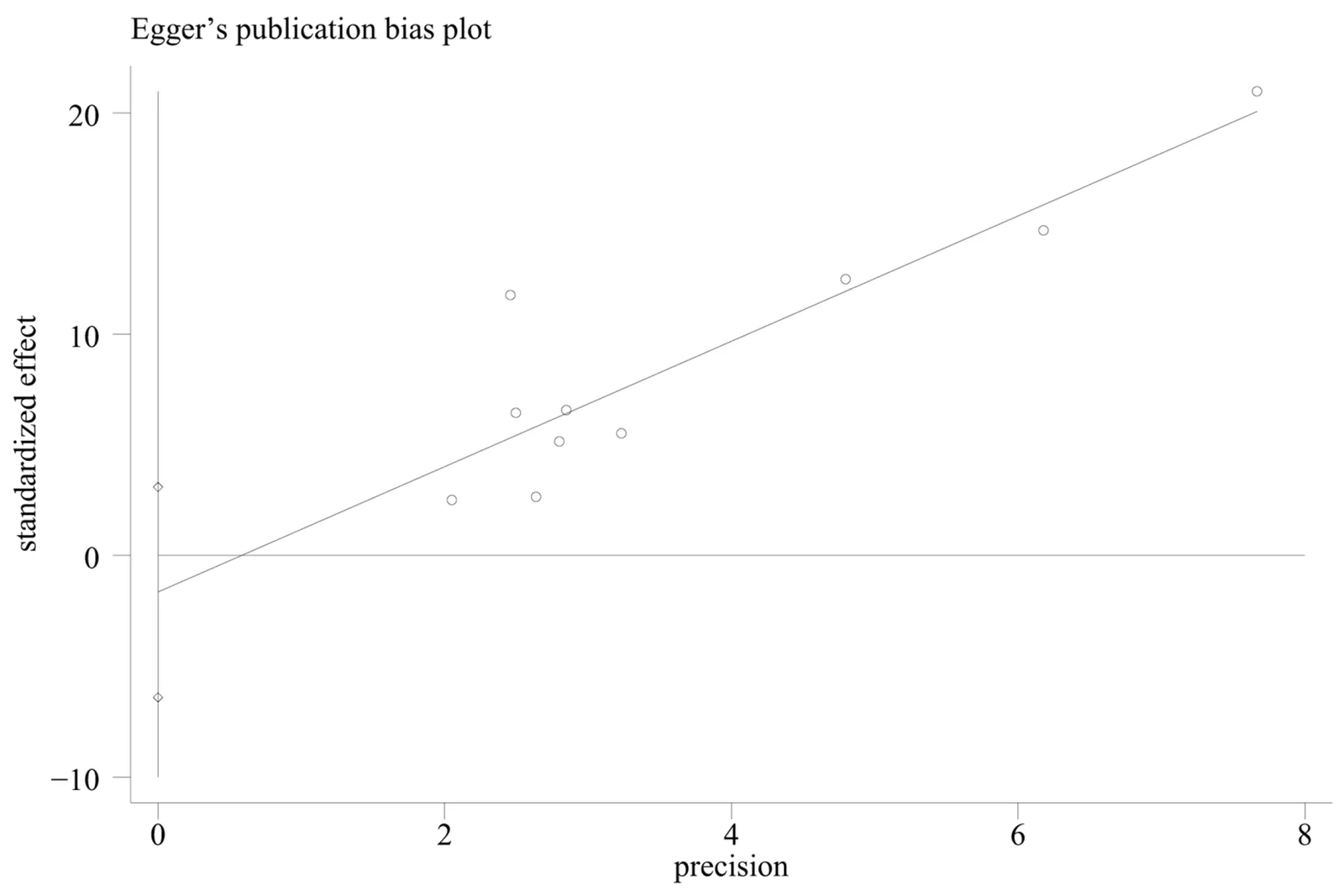
Publication bias at the clinical stage. Egger’s test for pathological grade (*p* = 0.443).

Begg’s and Egger’s tests were used to evaluate publication bias. In the pT staging, the *p* values of Begg’s test and Egger’s test were greater than 0.008 and 0.063, respectively (Figure 22 and Figure 23); in the pN staging, the *p* values of Begg’s test and Egger’s test were greater than 0.760 and 0.386, respectively (Figure 24 and Figure 25); in the pM staging, the *p* values of Begg’s test and Egger’s test were greater than 0.806 and 0.628, respectively (Figure 26 and Figure 27); in the AJCC staging, the *p* values of Begg’s test and Egger’s test were greater than 0.089 and 0.022, respectively (Figure 28 and Figure 29); in the tumor pathological grading, the *p* values of Begg’s test and Egger’s test were greater than 0.474 and 0.443, respectively (Figure 30 and Figure 31). There was publication bias in reports of pT and AJCC staging, but none for pN, pM staging, or tumor pathological grading.

**Figure 32:**
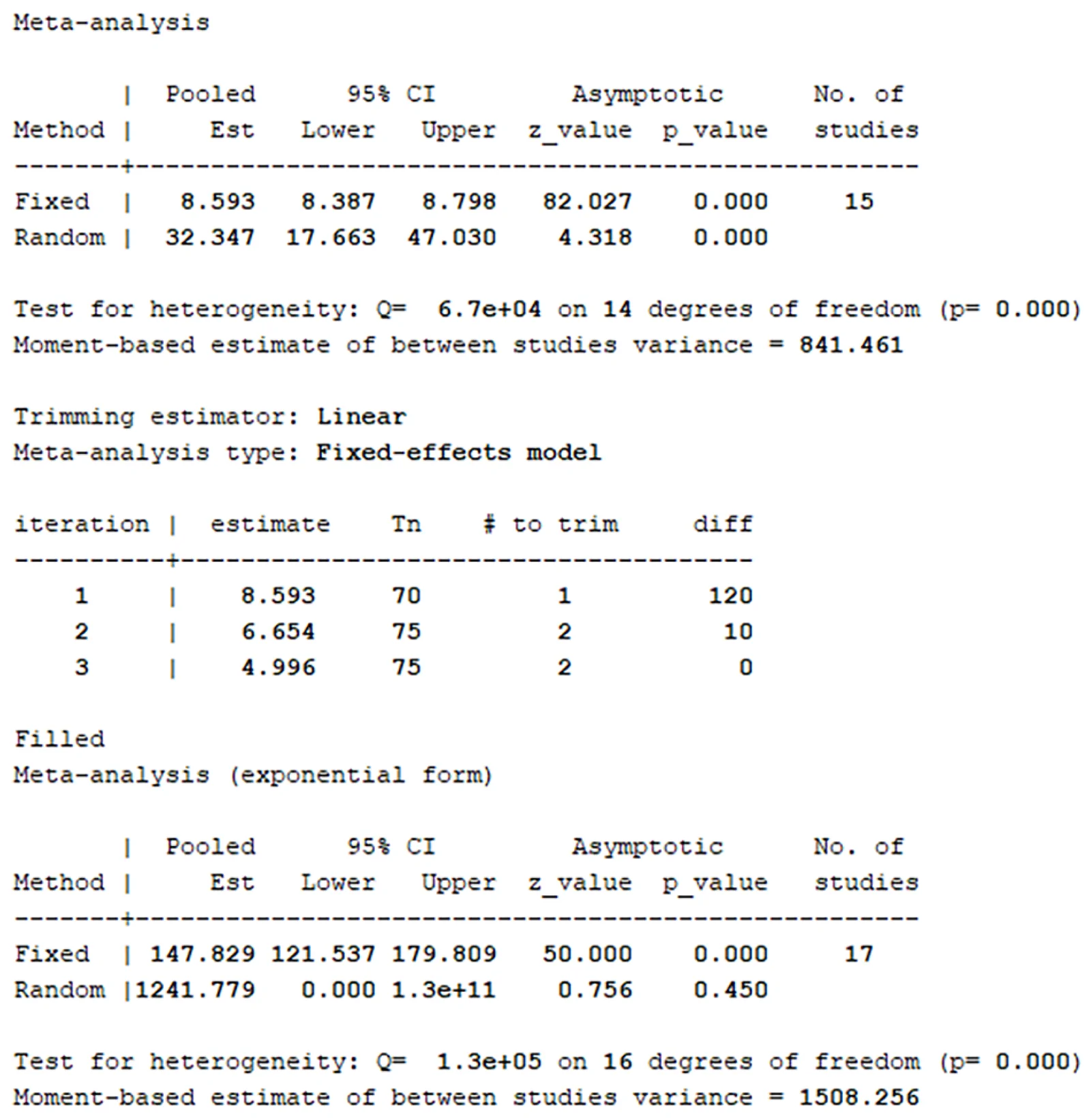
The results of the reduction and augmentation method in clinical staging. Trim-and-fill analysis for pT stage. After imputing two potentially missing studies, the pooled OR lost statistical significance (*p* changed from <0.001 to 0.450), indicating high sensitivity to publication bias.

**Figure 33:**
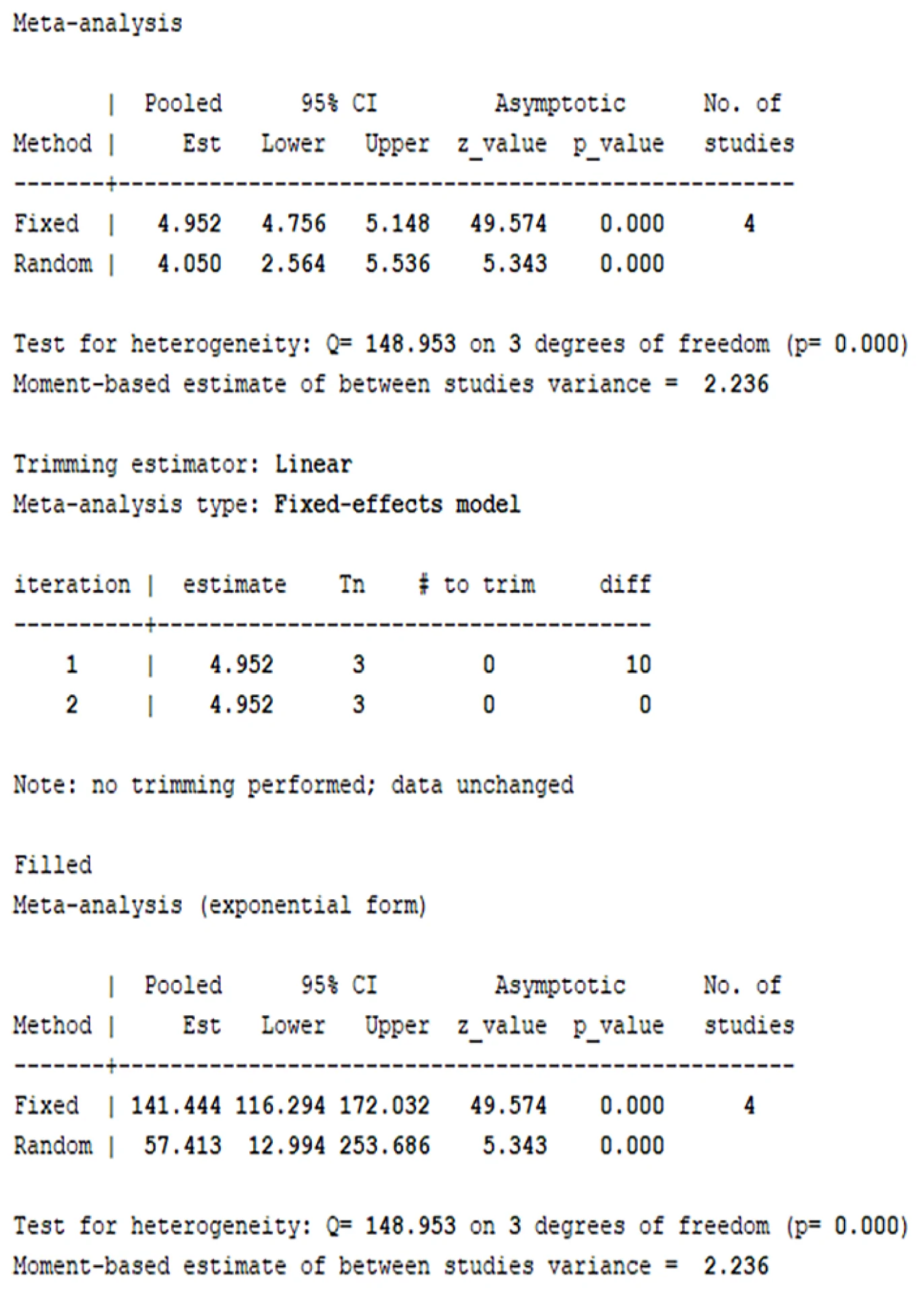
The results of the reduction and augmentation method for clinical staging. Trim-and-fill analysis for AJCC stage. With only four studies, the corrected estimate was unstable, and no reliable conclusion could be drawn.

To assess the impact of publication bias on the results, we used trim-and-fill analysis for correction. The analysis of pT staging indicated that after estimating and filling in two potentially missing studies, the combined results of the random effects model underwent a fundamental change; the statistical significance of the effect size disappeared (the *p* value changed from <0.001 to 0.450), and the confidence interval became extremely wide. This strongly suggests that the main findings of this meta-analysis are highly sensitive to potential publication bias, and the results are not robust. Therefore, the conclusion that SARIFA positivity is associated with high-grade pT staging should be viewed with caution (Figure 32). In the AJCC staging, since only four studies were included and there was significant publication bias and heterogeneity, the sensitivity analysis using the trim-and-fill analysis did not need to add any missing studies to make the funnel plot symmetrical. The estimation of the effect size was extremely unstable; so, no reliable conclusion could be drawn about the association between SARIFA positivity and tumor staging (Figure 33).

### 3.5. Study Selection

A list of studies excluded after full-text assessment, together with the specific reasons for exclusion, is provided in Supplementary Table S4 [24,25,26,27].

## 4. Discussion

This meta-analysis systematically evaluated the prognostic value of SARIFA as an emerging histopathological marker in solid tumors and its association with the development of high-grade tumors. The most robust finding was the consistent association between SARIFA positivity and worse overall survival, with very low heterogeneity (I^2^ = 0.0%) and no evidence of publication bias. This association remained stable across various subgroups, including cancer type, geographic region, definition used, statistical adjustment method, and data source. The pooled HR of 1.52 (95% CI: 1.42–1.63) indicates a 52% increase in mortality risk, and the lack of heterogeneity strengthens confidence in this estimate.

However, the evidence for clinicopathological parameters is more complex. Although SARIFA positivity was significantly associated with advanced pT (OR = 5.49), pN (OR = 2.80), pM (OR = 2.03), AJCC stage (OR = 4.04), and higher pathological grade (OR = 2.36) in pooled analyses, substantial heterogeneity and publication bias limits the credibility of these findings, particularly for pT and AJCC.

With regard to the sources of heterogeneity, differences between cancer types cannot be overlooked. The biological significance of a given stage category, such as T3–4 versus T1–2, may vary across tumor types because of differences in organ anatomy and the local adipose microenvironment. SARIFA is defined by tumor–adipocyte contact, so that its relevance inherently depends on the accessibility of local adipose tissue. For instance, colorectal cancer is surrounded by pericolonic fat, gastric cancer invades into the serosa or adjacent fat, prostate cancer is associated with periprostatic fat, and pancreatic cancer is embedded in peripancreatic fat, each of which provides a distinct context. Moreover, colorectal cancer invasion into pericolonic fat may be more readily captured by SARIFA than pancreatic cancer, which is often encased in a dense desmoplastic stroma. Consequently, SARIFA positivity is unlikely to represent an identical biological process across different organs. Furthermore, differences in the clinical thresholds of the TNM staging system across cancer types, particularly the AJCC stage, which integrates T, N, M, and cancer-specific rules, also contribute to heterogeneity. This is consistent with our subgroup analyses, which demonstrated that effect sizes varied by cancer type and staging parameter. For example, in both pN and pathological grade, the strongest associations were observed in colorectal cancer, followed by gastric cancer, whereas prostate and pancreatic cancers were not discussed in detail, due to the limited number of studies. In addition, most colorectal and gastric studies employed the conventional qualitative definition of SARIFA, whereas pancreatic and prostate studies adopted quantitative criteria. Although the definition-based subgroup analysis for overall survival did not reveal substantial differences, definitional inconsistency may still have introduced measurement heterogeneity for stage-related outcomes. Moreover, there were variations in study design and treatment. Most studies were retrospective, and differences in patient selection, treatment era, adjuvant therapy, follow-up duration, and pathological assessment protocols may also have affected both survival and staging estimates.

Despite the strong association observed in the pooled analysis between SARIFA positivity and advanced pT stage, the substantial heterogeneity (I^2^ = 87.4%) and the results of the sensitivity analysis suggest that this association is not robust and may be influenced by between-study differences and potential publication bias. In the trim-and-fill analysis, imputing only two studies changed the *p* value from <0.001 to 0.450, and the statistical significance was lost. Similarly, the AJCC analysis included only four studies and showed significant bias, indicating that the association between SARIFA and advanced AJCC stage should be regarded as hypothesis-generating rather than definitive. For pM, although the pooled OR was 2.03, heterogeneity (I^2^ = 70.5%) and the limited number of studies (only one each for colorectal and pancreatic cancer) preclude firm conclusions; this finding only suggests a possible association between SARIFA positivity and metastatic disease, without providing a definitive conclusion. In contrast, studies reporting pathological grade showed moderate heterogeneity (I^2^ = 31.7%) and no publication bias, suggesting a relatively reliable association with higher histological grade.

In colorectal and gastric cancers, SARIFA is defined as the presence of at least one tumor gland or ≥5 tumor cell clusters in direct contact with adipocytes at the invasive front, without intervening stromal reaction or inflammatory infiltration [3,6]. In prostate cancer and pancreatic ductal adenocarcinoma, quantitative criteria have been used for definition, and each of these two cancer types was represented by only one study [9,10]. The pathogenic mechanisms underlying SARIFA remain incompletely understood. Current evidence suggests that cancer cell proliferation is related to lipid metabolism; highly proliferative cancer cells increase lipid uptake and utilization [28], and proteins involved in fatty acid transport, such as CD36 and FABP4, are upregulated [26]. In addition, upregulation of urokinase-type plasminogen activator (uPA) and its inhibitor PAI-1 has been observed in SARIFA-positive cases. Since uPA and PAI-1 can devergrade the extracellular matrix, it has been hypothesized that this upregulation is linked to the direct contact between tumor cells and adipocytes in the absence of fibrous connective tissue in SARIFA-positive patients [26]. A reduction in natural killer cells has also been noted in SARIFA-positive cases, which may compromise antitumor immune function [29]. Collectively, these observations provide a biological rationale for the association between SARIFA positivity and more aggressive pathological features and poorer survival, which is generally consistent with the findings of the present meta-analysis.

This study is the first to provide a comprehensive quantitative assessment of the prognostic value of SARIFA and the risk of high-grade tumor development, with a large sample size and systematic evaluation of heterogeneity and bias. Nevertheless, several limitations should be acknowledged. First, the evidence for overall survival is relatively strong, and heterogeneity and bias associated with pathological grade were not significant; however, some stage-specific results, such as those for pT, exhibited substantial heterogeneity and publication bias, rendering them less robust. Second, there is a geographic limitation; 9 of the 14 included studies originated from Germany, and the remainder from other European countries, while evidence from Asia, North America, South America, and other regions is lacking. Although the regional subgroup analysis showed comparable HRs between Germany and other European countries, this finding should not be interpreted as evidence of geographic generalizability. Differences in ethnicity, healthcare systems, pathological practices, tumor epidemiology, and treatment patterns may limit the applicability of our findings to broader populations. Third, most studies were retrospective, with inherent risks of information bias and an inability to establish causality. Fourth, the evidence relating to pancreatic and prostate cancers is extremely limited, with only one study each; therefore, any conclusions regarding these cancer types should be considered preliminary and hypothesis-generating. Fifth, differences in SARIFA definitions across studies may have introduced measurement heterogeneity, although the OS subgroup analysis did not reveal major effects. Sixth, the lack of integration with molecular biomarkers, such as circulating tumor DNA (ctDNA), means that the incremental value of SARIFA beyond existing markers remains to be determined. Seventh, several limitations related to the review process should be acknowledged. Although a comprehensive search strategy was applied across multiple databases, potentially eligible studies may have been missed because unpublished studies and the gray literature were not systematically searched. Furthermore, the small number of available studies relating to several of the outcomes limited the ability to perform meta-regression and detailed subgroup analyses. Finally, because this review was based on aggregate rather than individual participant data, we were unable to perform patient-level analyses or uniformly adjust for potential confounding factors across studies.

Based on these findings, we consider that SARIFA status holds promise as a tool for clinicians to assess overall survival in cancer patients. Future research should focus on prospective study designs, expand investigations to cancer types beyond colorectal and gastric/oesophagogastric cancers, and further elucidate the underlying pathogenic mechanisms to develop novel targeted therapeutic strategies. In addition, an important direction for future research is to explore whether SARIFA provides complementary prognostic information to ctDNA. SARIFA is a tissue-based morphological marker that reflects local interactions between invasive tumor cells and the adipose microenvironment, whereas ctDNA is a dynamic liquid-biopsy measure of systemic tumor burden and molecular residual disease. Current evidence supports the prognostic relevance of ctDNA across various solid tumors, particularly in colorectal cancer, where postoperative or longitudinal ctDNA positivity is strongly associated with recurrence and survival [30]. However, we did not identify robust clinical studies directly evaluating the interaction between SARIFA and ctDNA. Therefore, prospective studies integrating SARIFA assessment with serial ctDNA measurements could determine whether these biomarkers provide independent or complementary prognostic information and whether their combination improves risk stratification beyond conventional TNM staging.

## 5. Conclusions

In conclusion, the available evidence supports SARIFA positivity as a promising prognostic marker associated with shorter overall survival, particularly in gastrointestinal cancers. However, the strength of evidence for its association with advanced pathological stage varies substantially. The associations with pT and AJCC stage are limited by considerable heterogeneity and potential publication bias, while evidence relating to pM is also heterogeneous. Moreover, the geographical concentration of studies in Europe and the very limited evidence from pancreatic and prostate cancers restrict the generalizability of these findings. SARIFA should therefore be regarded as a promising but not yet universally validated biomarker. Prospective multicenter studies integrating standardized SARIFA assessment with molecular and liquid-biopsy markers such as ctDNA are warranted.

## 6. Clinical Perspectives

Current prognosis assessment for cancer patients relies heavily on the TNM staging system, yet significant survival disparities exist among patients at the same stage, highlighting the urgent need for practical and reliable prognostic biomarkers. SARIFA, a novel histopathological marker assessable on routine H&E-stained sections, has shown preliminary promise but lacks systematic quantitative validation across multiple cancer types.

In summary, the results of this first comprehensive meta-analysis of 6881 patients across colorectal, gastric, pancreatic, and prostate cancers demonstrate that SARIFA positivity is significantly associated with a 52% increased risk of death (HR = 1.52, 95% CI: 1.42–1.63) and is strongly correlated with aggressive tumor phenotypes, including advanced pT, pN, pM, AJCC stage, and higher pathological grade.

Regarding its significance to human health and disease, as SARIFA assessment requires only conventional H&E staining without additional molecular or financial costs, this biomarker represents an immediately implementable, cost-effective tool for clinical risk stratification, potentially guiding personalized adjuvant treatment decisions and improving survival outcomes for millions of cancer patients worldwide.

## Figures and Tables

**Table 1:** Characteristics of the included studies [3,6,9,10,12,13,17,18,19,20].

First Author Published Year	Country	Cancer	Sample Size	Study Type	NOS Score	HR (95% CI) for OS	Analysis Method
Vilja V Tapiainen-Cohort 1-2025	Finland	CRC	1100	retrospective	8	1.58 (1.3–1.92)	multivariate analysis
Vilja V Tapiainen-Cohort 2-2025	Finland	CRC	776	prospective	8	1.48 (1.12–1.95)	multivariate analysis
Nic G Reitsam-2024	Germany	CRC	207	retrospective	7	2.5 (1.22–4.14)	multivariate analysis
Bianca Grosser-2022	Germany	Gastric/Esophagogastric cancer	480	retrospective	8	1.638 (1.153–2.326)	multivariate analysis
Nic G Reitsam-2025	Netherlands	CRC	1726	prospective	9	1.42 (1.24–1.63)	multivariate analysis
Bianca Grosser-MAGIC trial-surgery alone patients-2024	UK	Gastric/Esophagogastric cancer	158	retrospective	7	1.899 (1.285–2.806)	univariate analysis
Bianca Grosser-MAGIC trial-perioperative ECF patients-2024	UK	Gastric/Esophagogastric cancer	134	retrospective	7	1.4 (0.878–2.232)	univariate analysis
Bianca Grosser-ST03 trial-2024	UK	Gastric/Esophagogastric cancer	693	retrospective	8	1.974 (1.555–2.507)	multivariate analysis
Bianca Grosser-2023	Germany	Gastric/Esophagogastric cancer	489	retrospective	8	1.34 (1.03–1.7)	multivariate analysis
Johanna S Enke-2024	Germany	PCa	301	retrospective	7	1.29 (0.758–2.196)	univariate analysis;
Przemyslaw Grochowski-2024	Germany	PDAC	166	retrospective	8	1.637 (1.103–2.429)	multivariate analysis
Benedikt Martin-Cohort B-2021	Germany	CRC	253	retrospective	8	1.5 (0.9–2.5)	multivariate analysis
Kelly Offermans-2024	Netherlands	CRC	1727	prospective	9	1.46 (1.28–1.67)	multivariate analysis

CRC: Colorectal Cancer; PCa: Prostate Cancer; PDAC: Pancreatic Ductal Adenocarcinoma; NOS: the newcastle-ottawa quality assessment scale; OS: overall survival.

## Data Availability

The datasets analyzed in this meta-analysis are included within the article and its Supplementary Materials. No new raw clinical data were generated for this study. All relevant literature screening records and pooled statistical results are available in the Supplementary Files. Further inquiries can be directed to the corresponding author.

## Supplementary Materials

The following supporting information can be downloaded at: https://www.mdpi.com/article/10.3390/jcm15176830/s1, Table S1: PRISMA checklist; Table S2: Complete search strategies; Table S3: GRADE assessment of the certainty of evidence; Table S4: Studies excluded after full-text assessment and reasons for exclusion [24,25,26,27].
