# Supplementary material for: The Association of SARIFA with Poor Prognosis and Aggressive Tumor Phenotypes in Cancers: A Meta-Analysis
Source: J Clin Med. 2026 Sep 3;15(17):6830. doi: 10.3390/jcm15176830 (PMC13566555; doi:10.3390/jcm15176830)
Supplement: Supplementary file 1 [file jcm-15-06830-s001.zip › Table S2.pdf]

Supplementary Table S2. Complete search strategies

Database	Search strategy
PubMed	(SARIFA[Title/Abstract]) AND (("Prognosis"[Mesh]) OR (Prognoses[Title/Abstract] OR Prognostic Factors[Title/Abstract] OR Prognostic Factor[Title/Abstract] OR Factor, Prognostic[Title/Abstract] OR Factors, Prognostic[Title/Abstract]))
Web of Science	TS= (SARIFA) AND (Prognosis OR Prognoses OR Prognostic Factors OR Prognostic Factor OR Factor, Prognostic OR Factors, Prognostic)
Embase	(SARIFA:ti,ab) AND ('prognosis'/exp OR prognos*:ti,ab OR 'prognostic factor':ti,ab OR 'prognostic factors':ti,ab)
Cochrane Library	#1 SARIFA:ti,ab,kw; #2 MeSH descriptor: [Prognosis] explode all trees; #3 Prognosis:ti,ab,kw; #4 Prognoses:ti,ab,kw; #5 "Prognostic Factor":ti,ab,kw; #6 "Prognostic Factors":ti,ab,kw; #7 #2 OR #3 OR #4 OR #5 OR #6; #8 #1 AND #7
