# Supplementary material for: The Association of SARIFA with Poor Prognosis and Aggressive Tumor Phenotypes in Cancers: A Meta-Analysis
Source: J Clin Med. 2026 Sep 3;15(17):6830. doi: 10.3390/jcm15176830 (PMC13566555; doi:10.3390/jcm15176830)
Supplement: Supplementary file 1 [file jcm-15-06830-s001.zip › Table S3.pdf]

Supplementary Table S3. GRADE assessment of the certainty of evidence.

Outcome	Certainty
OS	Moderate
pT	Very low / Low
pN	Low
pM	Very low / Low
AJCC	Very low
Pathological grade	Low / Moderate
