# Supplementary material for: The Association of SARIFA with Poor Prognosis and Aggressive Tumor Phenotypes in Cancers: A Meta-Analysis
Source: J Clin Med. 2026 Sep 3;15(17):6830. doi: 10.3390/jcm15176830 (PMC13566555; doi:10.3390/jcm15176830)
Supplement: Supplementary file 1 [file jcm-15-06830-s001.zip › Table S4.pdf]

Supplementary Table S4. Studies excluded after full-text assessment and reasons for exclusion.

Author	Year	Reason for exclusion
Bianca Grosser	2024	review
Qingru Hu	2023	no extractable survival data
Bruno Märkl	2024	review
Mohamed J Saadh	2024	review
